# Serum reverse triiodothyronine in dogs with hypothyroidism, nonthyroidal illness syndrome and healthy dogs

**DOI:** 10.1371/journal.pone.0357106

**Published:** 2026-10-05

**Authors:** Julia Ruß, Ralf S. Mueller, Katharina Glos, Christina Meindl, Susanne Ritz, Daniel C. Müller, Stefanie Klenner-Gastreich, Christina Schwens

**Affiliations:** 1 Royal Canin GmbH & Co. KG, Köln, Germany; 2 Small Animal Clinic, LMU Munich, Munich, Germany; 3 Small Animal Centre Germering, Germering, Germany; 4 Anicura Veterinary Clinic Haar GmbH, Haar, Germany; 5 SYNLAB MVZ Labor München Zentrum GbR, Munich, Germany; 6 Antech Diagnostics Germany, Mars Petcare Science and Diagnostics, Viernheim, Germany; Universidade Federal de Minas Gerais, BRAZIL

## Abstract

**Background:**

Diagnosing canine hypothyroidism remains challenging because decreased total thyroxine (tT4) concentrations are not specific for hypothyroidism and are frequently observed in dogs with non-thyroidal illness syndrome (NTIS). Although additional thyrotropin (TSH) and free thyroxine (fT4) measurements may improve diagnostic accuracy, differentiation between hypothyroidism and NTIS remains difficult in clinical practice. In human medicine, reverse triiodothyronine (rT3), an inactive metabolite of thyroxine, has been proposed as a useful marker to distinguish hypothyroidism from NTIS. However, studies evaluating rT3 concentrations in dogs with hypothyroidism and NTIS are currently lacking.

**Objective:**

To establish and validate a canine-specific HPLC-MS/MS assay for reverse triiodothyronine (rT3), determine a reference interval in healthy dogs, and evaluate the diagnostic utility of rT3 for differentiating canine hypothyroidism from NTIS in comparison with free thyroxine measured by equilibrium dialysis (fT4d).

**Materials and methods:**

Reverse triiodothyronine (rT3) concentrations were measured by HPLC-MS/MS in healthy dogs, dogs with hypothyroidism, and dogs with NTIS. A canine-specific reference interval for rT3 was established using healthy adult dogs. Hypothyroidism was diagnosed based on compatible clinical signs, decreased total thyroxine (tT4), increased thyrotropin (TSH), and clinical improvement following levothyroxine supplementation. Dogs with moderate to severe systemic disease, decreased tT4 concentrations, and no clinical signs consistent with hypothyroidism were included in the NTIS group. The diagnostic value of rT3 in distinguishing between dogs with hypothyroidism and NTIS was investigated and compared with the diagnostic value of fT4 measured by equilibrium dialysis (fT4d) using ROC analysis.

**Results:**

47 healthy dogs, 18 hypothyroid dogs, and 23 dogs with NTIS were included. The reference interval for rT3 was 113–439 pg/ml. Median rT3 concentrations were significantly lower in hypothyroid dogs (2.5 pg/ml; [IQR 2.5–18.5 pg/ml], range 2.5–106 pg/ml) compared with dogs with NTIS (229 pg/ml; [IQR 148.7–538.3 pg/ml], range 52.2–700 pg/ml; p < 0.0001) and healthy dogs (281 pg/ml; [IQR 220–344.5 pg/ml], range 111–817 pg/ml; p < 0.0001). The area under the ROC curve (AUC) for rT3 to differentiate hypothyroidism from NTIS was 0.981 (95% CI: 0.951–1.010), compared with 0.896 (95% CI: 0.798–0.994) for fT4d. Using cut-off values of ≤52.2 pg/ml and ≤106 pg/ml, rT3 showed sensitivities and specificities of 83.4% (95% CI: 59–96%) and 100% (95% CI: 85–100%), and 100% (95% CI: 82–100%) and 82.6% (95% CI: 61–94%), respectively. Using the lower limit of the reference interval as a cut-off, sensitivity and specificity were 77.8% (95% CI: 53–92%) and 91.3% (95% CI: 72–98%).

**Conclusion and clinical relevance:**

The establishment of a reference interval for rT3 will enable future interpretation of rT3 values in dogs. rT3 represents a valuable diagnostic parameter for differentiating canine hypothyroidism from NTIS.

## Introduction

Hypothyroidism is one of the most common endocrine diseases in dogs but also one of the most over-diagnosed conditions in small animal practice, as low total thyroxine (tT4) without any other clinical or clinicopathological evidence of hypothyroidism is often used to treat dogs for hypothyroidism [1,2]. Diagnosing hypothyroidism by tT4 analysis is challenging as tT4 is not only low in hypothyroidism, but also in 30.2% up to 52.5% of dogs with non-thyroidal diseases [3–5].

For many years, thyrotropin (TSH) stimulation test was considered the gold standard among the blood tests to differentiate between hypothyroidism, non-thyroidal illness syndrome (NTIS) and healthy dogs [6–8]. Although recombinant human TSH is highly effective in differentiating hypothyroid from euthyroid dogs, even in cases with concurrent non-thyroidal disease or medication, its use in routine clinical practice is limited by high costs and restricted availability [8–10]. Scintigraphy is a further possibility to distinguish hypothyroid from healthy dogs [11,12]. Due to limited availability of this methodology and a mandatory general anaesthesia (although some protocols allow sedation [13,14]), this is not a suitable method for general veterinary practice [9]. A relatively recent diagnostic test to differentiate between hypothyroidism and NTIS in dogs is the TRH stimulation test with measurements of growth hormone (GH) and TSH plasma concentrations [15]. Unfortunately, the measurement of GH is not commercially available.

Free T4 (fT4), which represents approximately 1% of tT4, is considered to be the biologically active form of thyroxine that better reflects the true thyroid status [16]. FT4 can be measured by analogue methods (immunoassays or RIA; fT4a) or with equilibrium dialysis (fT4d) [3,4]. In general, fT4 measurement has a higher diagnostic specificity for canine hypothyroidism than tT4. Reported specificities range from approximately 97% for analogue assays (fT4a) to 90–100% for equilibrium dialysis methods (fT4d) [17]. However diagnostic sensitivity is not that high (80% fT4a and 71% up to 96% fT4d), measurement of fT4d is considered the single best diagnostic test for canine hypothyroidism as fT4d concentrations are less commonly influenced by drugs [17,18]. Furthermore, the measurement of fT4d is also unaffected by T4 autoantibodies [19]. In contrast to older studies that reported fT4 to be less influenced by NTIS [3] the study of Bennain showed values of fT4 under the reference interval in 89.1% (fT4a) and 57.1% (fT4d) in dogs with NTIS [3,19]. Unfortunately, the high cost and limited availability of equilibrium dialysis make fT4d measurement a second-line diagnostic test for hypothyroidism [9,18].

For this reason most veterinarians must rely on widely available hormone assays (generally tT4 and TSH), in combination with typical clinical signs of hypothyroidism to diagnose the disease [3,7,9]. Although decreased tT4 serum concentrations show a high sensitivity (90%) for diagnosing hypothyroidism, the specificity is low (82%) as non-thyroidal diseases as well as some drugs can also decrease tT4 concentrations, resulting in overdiagnosis of primary hypothyroidism in dogs [4,7,18,20]. Additional evaluation of TSH serum concentrations increases diagnostic specificity. Unfortunately, TSH shows a low sensitivity of only 57.7% up to 67% [21,22].

Reverse triiodothyronine (rT3) is an inactive metabolite of thyroxine (T4) produced by inner ring deiodination via deiodinase enzymes. In humans, decreased rT3 concentrations have been reported in hypothyroidism, whereas increased rT3 concentrations are observed in NTIS or stress as part of adaptive changes in thyroid hormone metabolism [23,24]. Measurement of rT3 has therefore been proposed as an additional parameter to differentiate hypothyroidism from NTIS in humans [25–30].

In dogs previous studies have reported rT3 measurements for investigations on the effects of anesthesia on thyroid function in which a significant perioperative increase in rT3 concentrations was observed [31]. Similarly, circulating rT3 concentrations have been evaluated in critically ill dogs with systemic inflammatory response syndrome, where no significant differences between affected and control dogs were identified [32]. More recently, an ultra-performance liquid chromatography–tandem mass spectrometry (UPLC-MS/MS) assay for the simultaneous quantification of total T4, T3, and rT3 in canine serum was analytically validated, and reference interval for healthy dogs were established [33]. However, no other studies evaluating rT3 specifically in dogs with hypothyroidism and NTIS were identified based on a broader search of PubMed, Wiley, and Google Scholar using the keywords “reverse T3,” “canine,” “dog,” and “thyroid”.

Therefore, this study aimed first to establish and validate an HPLC-MS/MS assay for measurement of rT3 in healthy dogs with determination of a reference interval, and second to evaluate whether rT3 is a useful parameter to differentiate between dogs with hypothyroidism and NTIS. A further aim was to compare rT3 with fT4 measured by equilibrium dialysis (fT4d). We hypothesized that rT3 would be able to differentiate hypothyroid from NTIS dogs and show comparable or complementary diagnostic performance to fT4d.

## Materials and methods

### Study design and general diagnostic workup

This study was conducted prospectively as a collaboration between a private veterinary clinic (Anicura Veterinary Clinic Haar), a university veterinary clinic (Small Animal Clinic of the LMU University of Munich) and Antech Lab Germany. All participating dogs were privately owned, the owners signed a written informed consent form, and all data were anonymised. The study was approved by the Ethics Committee of the Faculty of Veterinary Medicine/University of Munich under the number 253-12-25-2020. Recruitment of dogs took place between July 2021 and October 2023.

### Study population, inclusion and exclusion criteria

#### Healthy dogs – Reference interval calculation.

The reference interval of rT3 was calculated using healthy adult dogs (>one year), receiving no medical treatment, that were presented for an annual health assessment or as blood donor dogs. Dogs were included if general clinical examination, complete haematology, serum biochemistry and tT4 and TSH showed no abnormalities (deviations without clinical relevance of up to 10% above and below the reference interval were accepted as physiological). The dogs were recruited from the Anicura Veterinary Clinic Haar and the Small Animal Clinic of the University of Munich. Based on the criteria for establishing reference interval, in order to establish a robust reference interval, the sample size for the healthy dogs was set at a minimum of 40. Dog breeds with known differences in thyroid reference interval such as sled dogs and sighthounds were excluded from the study [9,34,35].

#### Dogs with hypothyroidism and NTIS – Diagnostic accuracy study.

To assess the diagnostic value of rT3, dogs with hypothyroidism were compared with those with NTIS. The diagnosis of hypothyroidism was established based on at least two typical clinical signs (lethargy, weight gain, cold intolerance, alopecia, seborrhoea, bacterial pyoderma or ear infections) [9,16,36] in combination with low tT4 and elevated TSH serum concentrations. Furthermore, a clinical response to supplementation of levothyroxine after diagnosis of hypothyroidism with concurrent normalisation of the tT4 serum concentration was a requirement for inclusion.

Dogs with NTIS had a known moderate to severe underlying disease as defined by Kantrowitz [3] and showed a decreased tT4 concentration with normal or increased TSH [37]. Hypothyroidism was ruled out in all dogs based on a combination of history, clinical assessment and laboratory findings and the underlying disease was considered sufficient to explain the altered thyroid parameters.

The dogs with hypothyroidism were either referred to the Anicura Veterinary Clinic Haar or were recruited via veterinarians submitting samples to Antech Lab Germany. Only dogs with a decreased tT4 and elevated TSH were included, while others were excluded. All dogs with suspected NTIS were initially screened at the Anicura Veterinary Clinic Haar; only those dogs with a decreased tT4 concentration were included in the study. The target group size was determined a priori based on a statistical power calculation using a sample size calculator (https://clincalc.com/stats/samplesize.aspx, last accessed 29.7.2026). With a power of 0.8, a significance level of 0.05 and a rT3 standard deviation of 130, 15 dogs were needed to identify a difference of 120 in the mean values of rT3 between groups. For this reason a target group size of at least 15 dogs per group was chosen.

As for the healthy dogs, sled dogs and sighthounds were excluded from the study, as were dogs receiving drugs known to influence fT4 concentrations such as glucocorticoids, phenobarbital, sulfonamides, carprofen or clomipramine [9,34,35]. Only ongoing treatments were considered for exclusion; no predefined discontinuation period for prior medication was applied, as the study population included both dogs with acute conditions and dogs with chronic diseases receiving previous treatment.

### Laboratory testing

In the healthy control population, four millilitres of whole blood and one millilitre of EDTA blood was taken. In all dogs with suspected hypothyroidism and non-thyroidal diseases, blood samples of eight millilitres of whole blood and at least one millilitre of EDTA blood were obtained. The blood sample was taken in all dogs either from the cephalic vein or the lateral saphenous vein. After blood collection, serum was allowed to equilibrate at room temperature for approximately 20 minutes prior to centrifugation. EDTA and two millilitres of serum were stored under refrigeration at 5℃ and sent to Antech Lab Germany and SYNLAB MVZ Labor München Zentrum GbR the same day. For all hypothyroid dogs and dogs with NTIS additionally two millilitres of serum were frozen at −18℃ and sent frozen to the Veterinary Diagnostic Laboratory (VDL) at Michigan State University.

In all participants (healthy dogs, dogs with hypothyroidism or NTIS), a complete blood count (ADVIA 2021 – Siemens), serum biochemistry profile (AU5800 Chemistry Analyzer – Beckman Coulter), tT4, and TSH were measured by Antech Lab Germany. The following analytes were included in the serum biochemistry profile: albumin, globulin, total protein, bilirubin, triglyceride, cholesterol, glucose, fructosamine, urea, creatinine, alkaline phosphatase (AP), aspartate aminotransferase (AST), alanine aminotransferase (ALT), glutamate dehydrogenase (GLDH), gamma glutamyltransferase (µGT), amylase, lipase (DGGR), creatinine kinase (CK), calcium, phosphate, magnesium, sodium, potassium, chloride.

Serum tT4 and TSH concentrations were measured using a chemiluminescent enzyme immunoassay (IMMULITE 2000, Siemens, canine tT4 and TSH assays) validated for dogs. Laboratory specific reference intervals were 1.5–4.5 µg/dl (tT4) and <0.59 ng/ml (TSH). The laboratory specific lower limit of quantification of tT4 was 0.12 µg/dl and was determined in accordance with the ASVCP quality assurance guidelines [38]. The laboratory specific coefficients of variation (CV%) of the intra- and interassay precision for tT4 are 7.8%, 7.0% and 3% and 7.7% and 4.16% at 1.23, 3.29 and 5.18 µg/dl (intraassay) and 1.03 and 2.26 µg/dl (interassay). As reported by the manufacturer the lowest detectable concentration of TSH was 0.03 ng/ ml. The intraassay and interassay CVs were 5.0%, 4.7%, and 5.7% and 10%, 5.5%, and 6.1% at TSH concentrations of 0.2, 2.35 and 6.1 ng/ml respectively.

All three groups of dogs had rT3 measurements performed at SYNLAB MVZ Labor München Zentrum GbR using HPLC-MS/MS. In the dogs with hypothyroidism and NTIS additional thyroid parameters were evaluated by the Veterinary Diagnostic Laboratory (VDL) at Michigan State University to further characterize thyroid status and support the differentiation between hypothyroidism and NTIS: total triiodothyronine (tT3), fT4d, T4 autoantibody (T4AA), T3 autoantibody (T3AA) and thyroglobulin autoantibody (TgAA). Serum tT3 was measured by RIA with a reference interval of 0.5–1.4 ng/ml. Serum fT4d concentration was measured after equilibrium dialysis (ED) using a radioimmunoassay (RIA) with a reference interval of 0.5–3.3 ng/dl. TgAA were measured by ELISA having a reference interval of 0–35%. T3AA and T4AA were both measured by RIA with a reference interval of 0–20% (T3AA) and 0–10% (T4AA).

### Statistical analysis

The statistical analyses were performed using Abacus 3.0 and Analyse it 5.30. The reference interval for rT3 in healthy dogs was calculated using a robust untransformed method based on the Guidelines for the Determination of de novo reference intervals in veterinary species and other related topics provided by the American Society for Veterinary Clinical Pathology (ASVCP) [39]. Prior to the calculation the dataset was evaluated for potential outliers using Tukey’s method. The Anderson-Darling test was used to assess the normality of the distribution of age, weight and all analyzed laboratory data (tT4, TSH and rT3) within the 3 groups. Normally distributed data (weight) were tested for significance using ANOVA and the Tukey test for multiple comparisons, whilst non-normally distributed data (age, tT4, TSH, rT3) were tested using the Kruskal-Wallis test followed by the Steel-Dwass-Critchlow-Fligner test for multiple comparisons. Normally distributed data were reported as mean ± standard deviation (SD) (weight) whilst pairwise results are presented as mean difference with corresponding 95% confidence interval. Non-normally distributed data are shown as medians with interquartile ranges [first quartile: Q1, third quartile: Q3] and all pairwise differences are reported as Hodges-Lehmann location shift with 95% confidence intervals. In healthy dogs, rT3 values were compared between young (1–6 years) and old dogs (7–12 years) using Wilcoxon-Mann-Whitney test after testing for normality. Furthermore, the association of age and rT3 was determined using Spearman’s rank correlation. Sensitivity and specificity of rT3 and fT4d for comparison of hypothyroid dogs and NTIS dogs were evaluated using receiver operating characteristic (ROC) analysis. RT3 values below the detection limit of 5 were reported as 2.5 as done in other comparable studies [40]. Statistical significance was defined as p < 0.05.

## Results

### Study population

#### Healthy dogs and reference interval.

Of 56 eligible healthy dogs, 9 had to be excluded due to insufficient sample material (n = 2) or due to low tT4 concentration (n = 7). Of the 47 dogs that were considered healthy by clinical and laboratory examination, 24 were recruited via Anicura Veterinary Clinic Haar and 23 by the Small Animal Clinic of the University of Munich (Fig 1).

**Fig 1:**
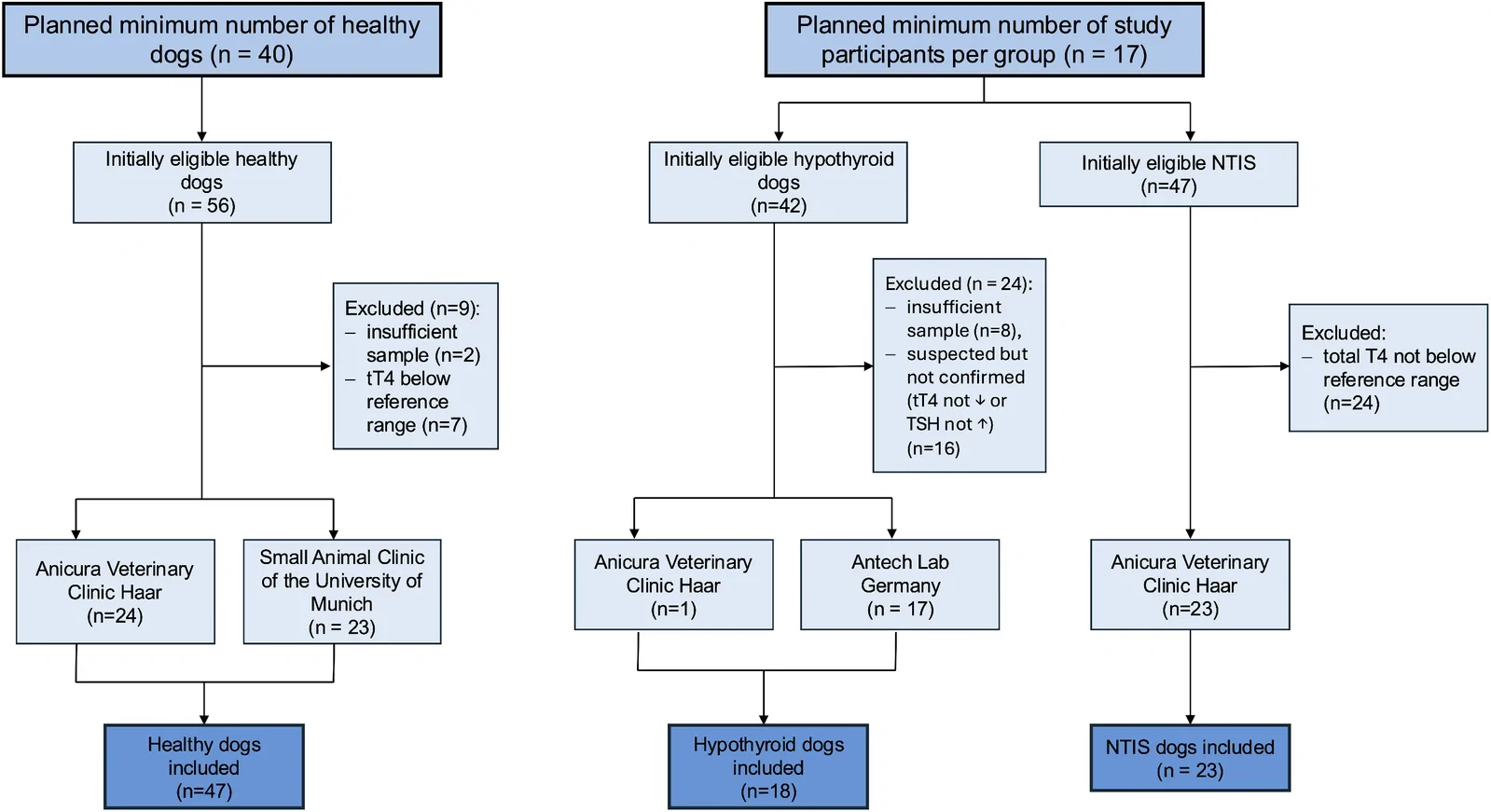
Study flow diagram of dog inclusion and exclusion across groups. Initially eligible dogs were categorized as healthy (n = 56), hypothyroid (n = 42), or suspected NTIS (n = 47). Exclusion criteria included insufficient sample material and biochemical findings inconsistent with group definitions (e.g., tT4 within reference interval or lack of TSH elevation). After exclusions, 47 healthy dogs, 18 hypothyroid dogs, and 23 NTIS dogs were included in the final analysis.

The range of rT3 in the 47 healthy dogs was 111–817 pg/ml (median 281 pg/ml [IQR 220–344.5 pg/ml]) (Table 1). RT3 concentrations differed significantly between younger (1–6 years, n = 35) and older (7–12 years, n = 12) healthy dogs, with higher values observed in younger dogs (median 316 pg/ml [IQR 247–350 pg/ml]) compared to older dogs (median 232 pg/ml [IQR 184–283 pg/ml]; p = 0.0065) (Fig 2). Spearman’s rank correlation analysis showed a moderately (p = 0.4–0.6) negative correlation (Spearman’s p = −0.472 [95% CI: –0.674 to –0.206]) between rT3 and age in dogs.

**Table 1:** Baseline and laboratory characteristics.

Parameter (Reference interval)	Healthy (n = 47)	Hypothyroid (n = 18)	NTIS (n = 23)
**tT4 ((1.5–4.5 µg/dl)**	2.3 [IQR 1.8–2.6]	0.1 [IQR 0.1–0.3]	0.1 [IQR 0.2–1.1]
**TSH (< 0.59 ng/ml)**	0.1 [IQR 0.1–0.2]	2.3 [IQR 1.3–6.2]	0.2 [IQR 0.1–0.2]
**fT4 (0.5–3.3 ng/dl)**	*not measured*	1.5 [IQR 1.0–5.1]	10 [IQR 7.2–14.0]
**rT3 (113–439 pg/ml)**	281 [IQR 220.2–344.5]	2.5 [IQR 2.5–18.5]	229 [IQR 148.7–538.3]

**Fig 2:**
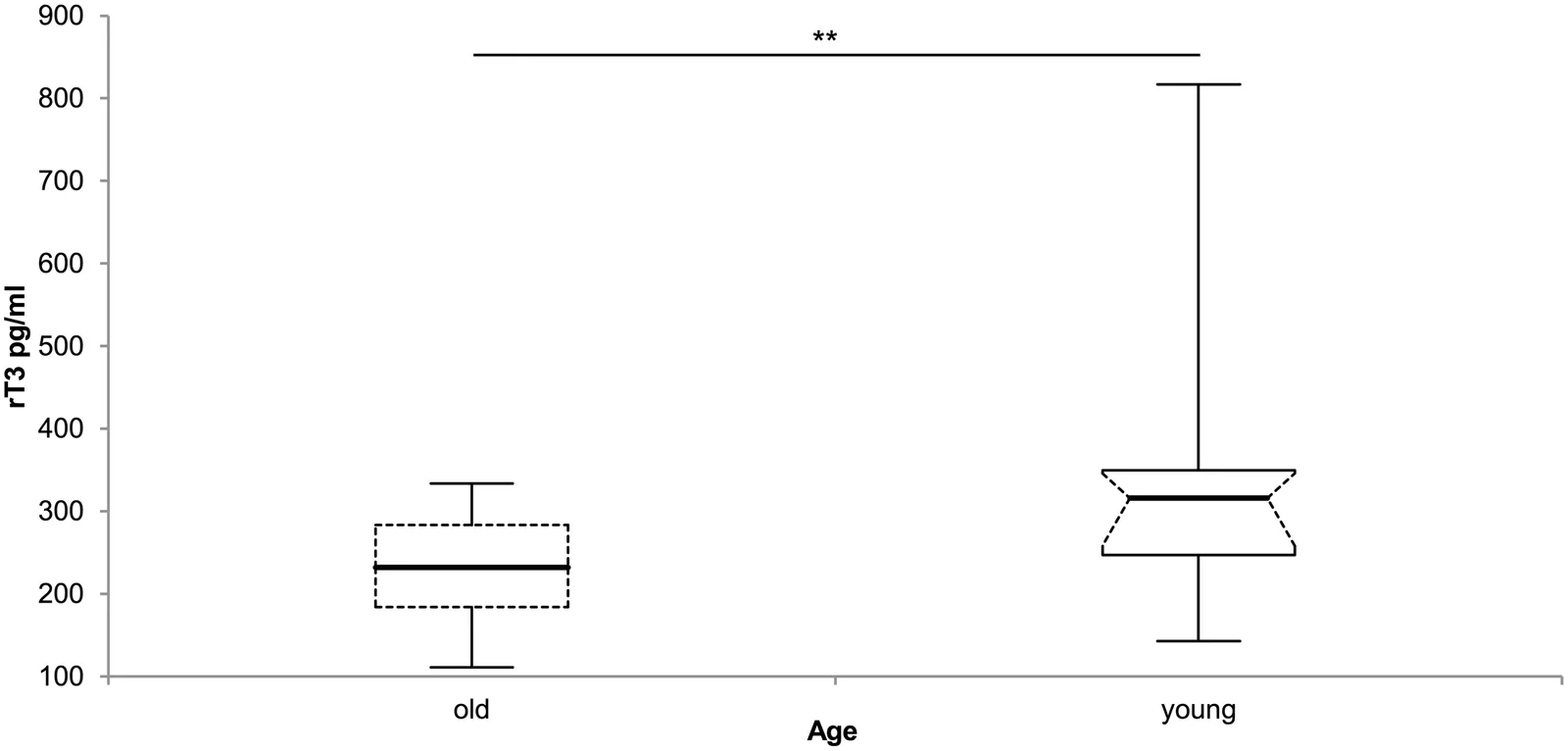
Association between age and serum rT3 concentrations in healthy dogs. Box-and-whisker plot showing the age distribution of rT3 levels in young (1–6 years) and old (7–12 years) dogs. The box represents the interquartile range (IQR) between the first quartile (Q1) and the third quartile (Q3), containing the central 50% of the data, while the line inside the box indicates the median. The whiskers extend to the minimum and maximum observed values. Young dogs had significantly lower rT3 concentrations compared to older dogs (**p = 0.0065).

For the calculation of the reference interval in healthy dogs, three outliers (586, 616, and 817 pg/ml) were identified and excluded. For the remaining 44 healthy dogs, the reference interval for rT3 was 113–439 pg/ml [39].

#### Hypothyroid dogs and dogs with NTIS.

42 dogs suspected of having hypothyroidism were initially included in the study. Of these dogs, 24 were excluded because there was insufficient sample material (n = 8) or because the dogs did not meet the necessary inclusion criteria (low tT4 and elevated TSH) (n = 16). Of the 18 dogs ultimately included in the study, one was recruited via the Anicura Veterinary Clinic in Haar and the other 17 via Antech Lab Germany.

Of the 47 dogs initially examined for NTI, 24 were excluded as they did not have low tT4 levels. All 23 dogs with a final diagnosis of NTIS were diagnosed at the Anicura Veterinary Clinic in Haar (Fig 1).

#### Age and Weight Correlation (Healthy Dogs, Hypothyroid dogs, Dogs with NTIS).

Hypothyroid dogs ranged in age from two to 10 years (median: 5.5 years [IQR 4–8]) and in weight from four to 59 kg (mean: 26.3 kg ± 14.3 kg). There were six females (five spayed) and 12 males (five neutered). The group of 23 dogs with NTIS ranged from one to 15 years (median: nine years [IQR 6.2–12]) and weighed from 6.5 to 42.5 kg (mean: 24.5 kg ± 11.3). Dogs with NTIS consisted of 11 females (five spayed) and 12 males (six neutered). The 47 healthy dogs ranged in age from one to 12 years (median: five years [IQR 3–6.8]) and in weight from two to 45 kg (mean: 21.9 kg ± 9.3). There were 24 females (13 spayed) and 23 males (11 neutered) (Table 2).

**Table 2:** Baseline characteristics of healthy dogs, dogs with hypothyroidism and NTIS.

Parameter	Healthy (n = 47)	Hypothyroid (n = 18)	NTIS (n = 23)
Median age (years)	5 ([IQR 3–6.8], range: 1–12)	5.5 ([IQR 4–8], range 2–10)	9 ([IQR 6.2–12], range 1–15)
Mean body weight (kg)	21.9 ± 9.3, range 2–45	26.3 ± 14.3, range 4–59	24.5 ± 11.3, range 6.5–42.5

Baseline characteristics, including breed, age, sex, neuter status, and body weight, as well as thyroid-related parameters (TSH, tT4, and rT3), are provided in S1–S3 Tables in the Supporting Information. In hypothyroid and NTIS dogs, additional thyroid parameters including fT4d and further thyroid function markers are included. Disease-specific clinical information is additionally provided for hypothyroid dogs (clinical signs) and NTIS dogs (underlying diseases).

Dogs with NTIS were significantly older than dogs with hypothyroidism (Hodges-Lehmann location shift: 3, 95% CI 0–6, p = 0.04) and healthy dogs (Hodges-Lehmann location shift: 4, 95% CI 2–6, p = 0.002). No significant age difference was observed between hypothyroid and healthy dogs (Hodges-Lehmann location shift: 1, 95% CI −1–3, p = 0.5) (Table 3, Fig 3). There was no significant difference in body weight among the groups (Table 4).

**Table 3:** Pairwise comparisons of age between healthy dogs, dogs with hypothyroidism, and dogs with nonthyroidal illness syndrome (NTIS).

Comparison	Hodges–Lehmann location shift (years)	95% CI	P-value
NTIS vs. Hypothyroid	3	0–6	0.040
NTIS vs. Healthy	4	2–6	0.002
Hypothyroid vs. Healthy	1	−1–3	0.500

**Table 4:** Pairwise comparisons of body weight between healthy dogs, dogs with hypothyroidism, and dogs with nonthyroidal illness syndrome (NTIS).

Comparison	Mean difference (kg)	95% CI	P-value
Hypothyroid vs. Healthy	4.4	−3.0 to 11.7	0.34
Hypothyroid vs. NTIS	1.8	−6.5 to 10.1	0.87
NTIS vs. Healthy	2.6	−4.0 to 9.2	0.62

**Fig 3:**
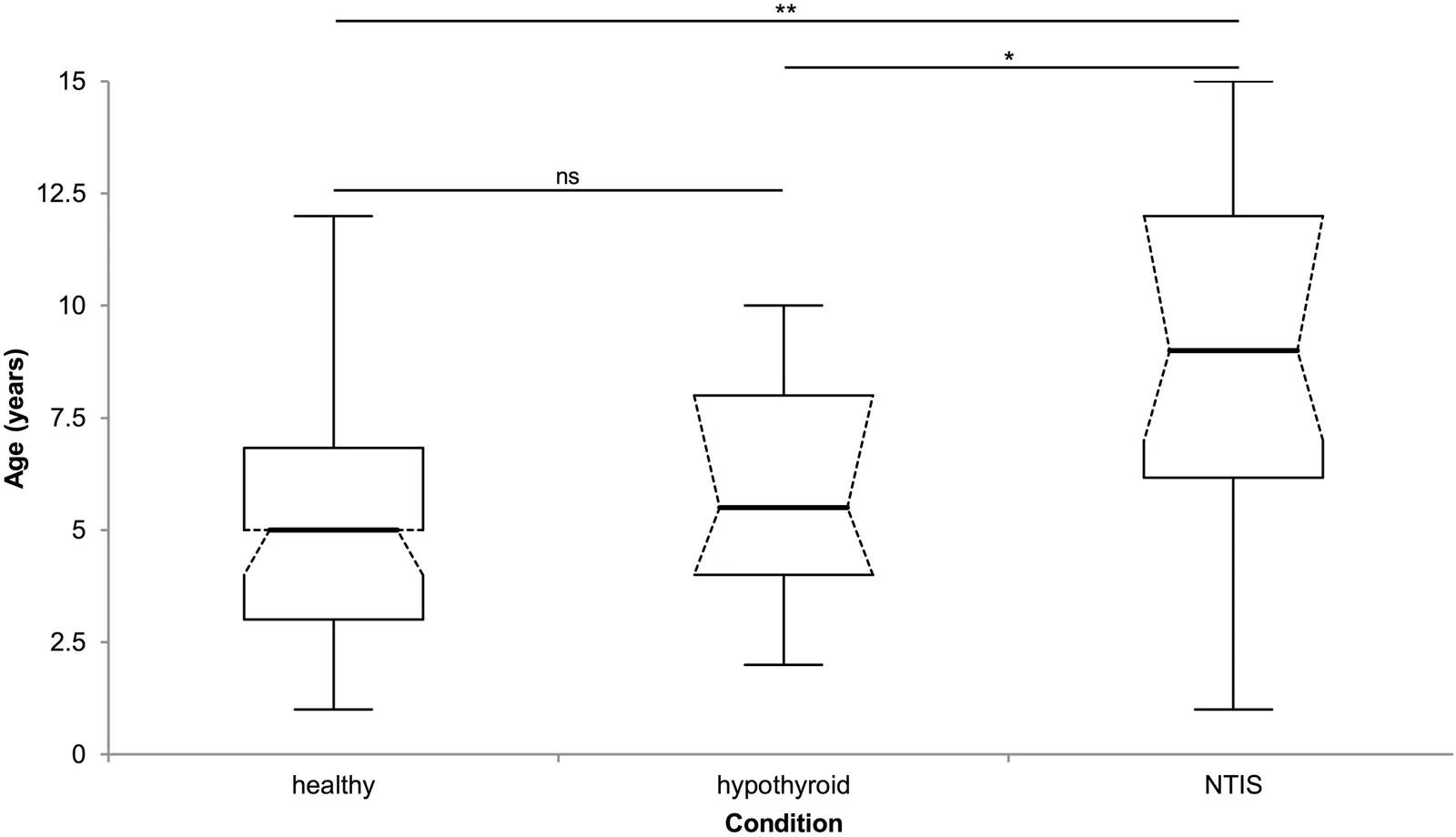
Age distribution of the three study groups (hypothyroid dogs, NTIS and healthy controls). Box-and-whisker plot showing the age distribution across the three groups of dogs studied (healthy, hypothyroid and NTIS). Dogs with NTIS were significantly older than hypothyroid (*p = 0.04) and healthy dogs (**p = 0.002), whereas no significant age difference was observed between hypothyroid and healthy dogs (ns p = 0.5).

### Validation of rT3 assay

The HPLC-MS/MS validated method of measuring rT3 was routinely applied to human serum samples. The linearity was found to be between 5–700 pg/ml. Intra- and interassay accuracy and precision were tested at two different levels (n = 10) showing a coefficient of variation (CV) of 2% (rT3 182 pg/ml), 3.9% (rT3 444 pg/ml) and 6.4% (rT3 182 pg/ml), 6.3% (rT3 444 pg/ml) for human samples. Additionally, intraassay precision was investigated in two different dog serum samples. The CV was 5.5% at 159 pg/ml (n = 6) and 2.3% at 372 pg/ml (n = 8). Stability of canine pooled serum samples was found to be 7 days at room temperature, 14 days at 2–8 °C and at least 3 weeks at ≤–18 °C.

#### Comparison of rT3 in dogs with hypothyroidism, NTIS and healthy dogs.

Hypothyroid dogs exhibited rT3 values ranging from 2.5 to 106 pg/ml (median 2.5 pg/ml [IQR 2.5–18.5 pg/ml]), with 13/18 dogs (72.2%) showing values of 2.5 pg/ml. Dogs with NTIS showed rT3 values ranging from 52.2 to 700 pg/ml (median 229 pg/ml [IQR 148.7–538.3 pg/ml]). RT3 concentrations were significantly lower in hypothyroid dogs compared to dogs with NTIS (Hodges-Lehmann location shift: 225.5, 95% CI 137.4 to 452.5, p < 0.0001) and healthy dogs (Hodges-Lehmann location shift: 257.5, 95% CI 215.5 to 318.5, p < 0.0001). No statistically significant difference in rT3 concentrations was observed between healthy and NTIS dogs (Hodges-Lehmann location shift: 51, 95% CI – 55–123, p = 0.39) (Fig 4, Table 1, Table 5).

**Table 5:** Pairwise statistical comparisons between healthy, hypothyroid, and NTIS dogs (Hodges-Lehmann location Shift (95% CI), p-values).

Parameter (Reference Interval)	Healthy vs Hypothyroidism	Healthy vs NTIS	Hypothyroidism vs NTIS
tT4 (1.5–4.5 µg/dl)	2.08 (1.72 to 2.38), p < 0.0001	1.64 (1.34 to 2.02), p < 0.0001	0.31 (0.02 to 0.86), p = 0.0084
TSH (< 0.59 ng/ml)	2.09 (1.57 to 5.53), p < 0.0001	0 (−0.06 to 0.07), p = 0.99	2.02 (1.29 to 5.6), p < 0.0001
rT3 (113–439 pg/ml)	257.5 (215 to 318.5), p < 0.0001	51 (- 55–123), p = 0.39	225.5 (137.4 to 452.5), p < 0.0001

**Fig 4:**
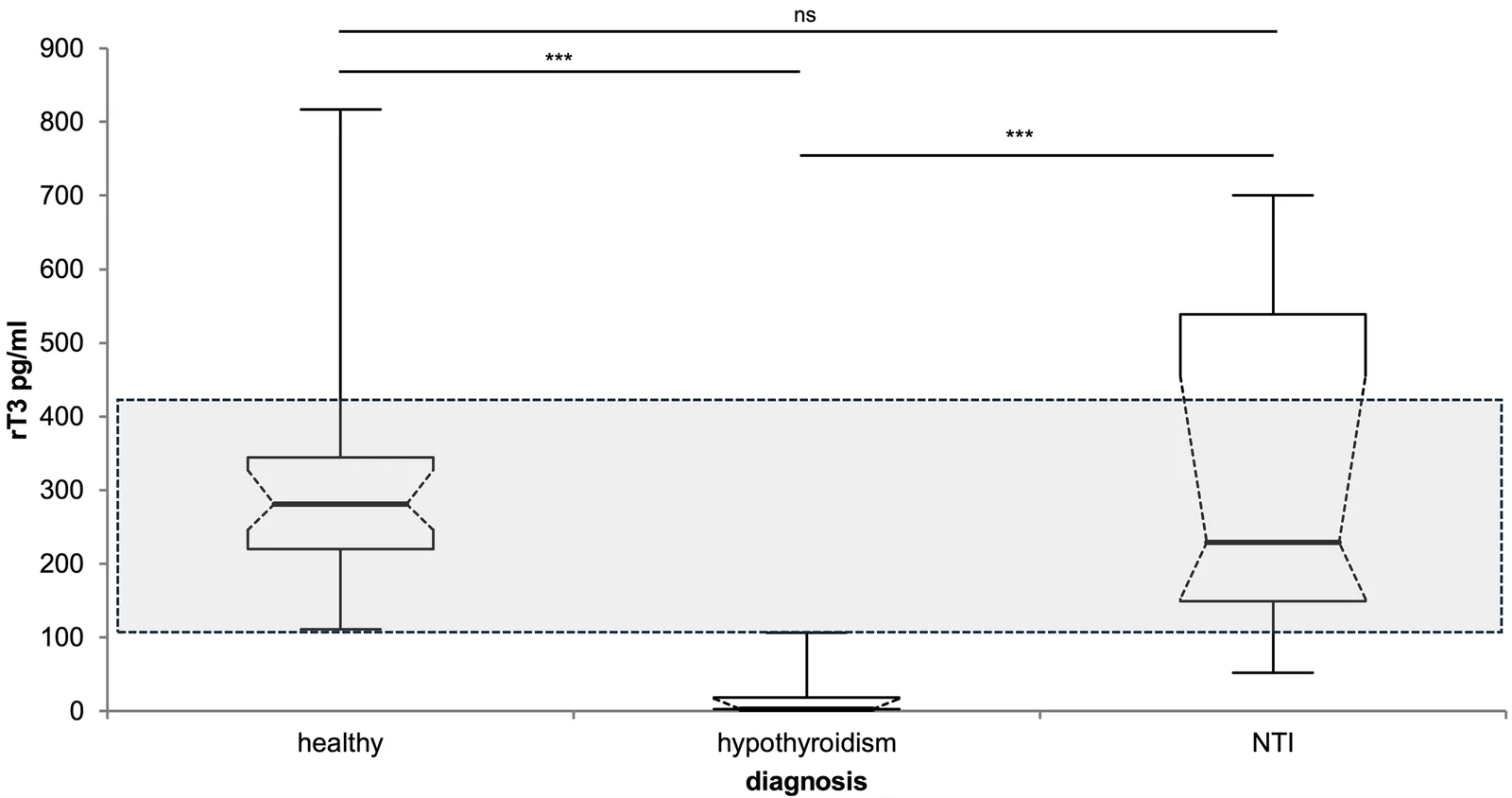
Serum reverse triiodothyronine (rT3) concentrations in healthy dogs, dogs with hypothyroidism, and dogs with NTIS. Box-and-whisker plot showing the rT3 distribution across the three groups of dogs (healthy, hypothyroid and NTIS). The box represents the interquartile range (IQR) between the first quartile (Q1) and the third quartile (Q3), containing the central 50% of the data, while the line inside the box indicates the median. The whiskers extend to the minimum and maximum observed values. The gray shaded background indicates the reference interval derived from the healthy control group (113–439 pg/ml). Dogs with hypothyroidism showed significantly lower rT3 concentrations compared to dogs with NTIS (***p < 0.0001) and healthy dogs (***p < 0.0001). No significant difference in rT3 concentrations was observed between healthy dogs and dogs with NTIS (ns p = 0.39).

#### Diagnostic performance of rT3 and fT4d.

For rT3, the area under the curve (AUC) for the differentiation of dogs with hypothyroidism and NTIS was 0.981 (95% CI: 0.951–1.010). A cut-off value of 52.2 pg/ml yielded a sensitivity of 83.4% (95% CI: 59–96%) and a specificity of 100% (95% CI: 85–100%). rT3 values >106 pg/ml were exclusively observed in dogs with NTIS resulting in a sensitivity of 100% (95% CI: 82–100%) and a specificity of 82.6% (95% CI: 61–94%) when choosing a cut-off of 106 pg/ml. A cut-off value of 99 pg/ml provided the best balance between sensitivity (94.4%; 95% CI: 73–99%) and specificity (87%; 95% CI: 66–97%) (Fig 5, Table 6).

**Table 6:** Diagnostic performance of different rT3 cut-off values for differentiating hypothyroidism from NTIS.

rT3 cut-off (pg/mL)	Interpretation	Sensitivity % (95% CI)	Specificity % (95% CI)
52.2	Best specificity	83.4 (59–96)	100 (85–100)
99	Best balance (Youden index)	94.4 (73–99)	87.0 (66–97)
106	Best sensitivity	100 (82–100)	82.6 (61–94)

**Fig 5:**
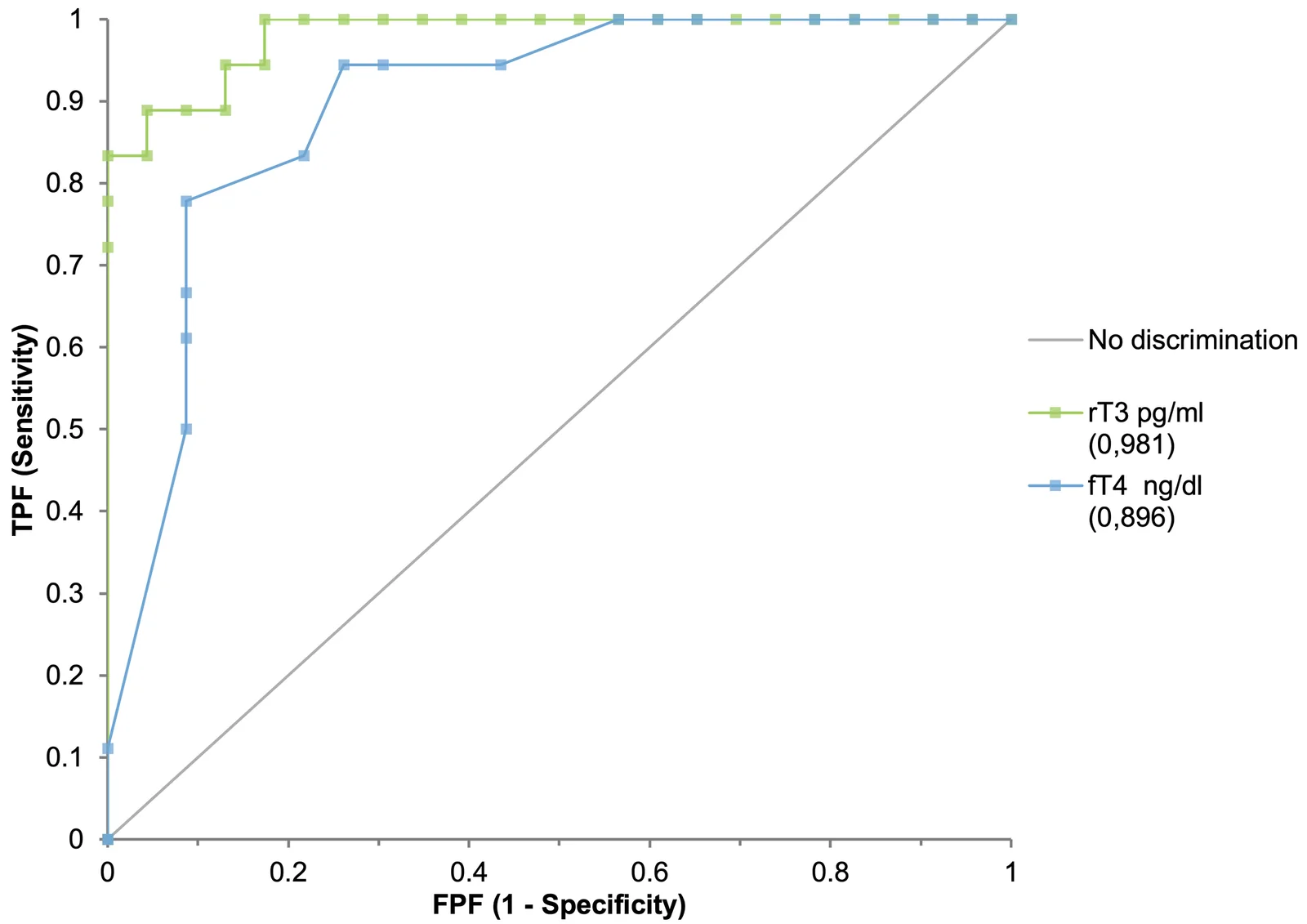
ROC analysis comparing the diagnostic performance of rT3 and fT4d for differentiation between hypothyroid dogs (n = 18) and dogs with NTIS (n = 23). The AUC for rT3 was 0.981 (95% CI: 0.951–1.010), indicating superior diagnostic performance compared to fT4d (AUC 0.896; 95% CI: 0.798–0.994). RT3 values ≤52.2 pg/ml showed a specificity of 100% (95% CI: 85–100%) with a sensitivity of 83.4% (95% CI: 59–96%) for confirming hypothyroidism, while a cut-off ≤106 pg/ml achieved a sensitivity of 100% (95% CI: 82–100%) with a specificity of 82.6% (95% CI: 61–94%). For fT4d, the lower reference limit cut-off of 0.46 ng/dl resulted in a sensitivity of 77.8% (95% CI: 53–92%) and specificity of 91.3% (95% CI: 72–98%). A highly specific cut-off of 0.08 ng/dl yielded a specificity of 100% (95% CI: 85–100%) but low sensitivity (11%; 95% CI: 3–32%), whereas a cut-off of ≤0.85 ng/dl achieved 100% sensitivity (95% CI: 82–100%) with a specificity of 43.5% (95% CI: 24–64%).

For fT4d, the AUC curve for distinguishing between the two groups was 0.896 (95% CI: 0.798–0.994) (Fig 5). Using the lower limit of the reference interval (0.46 ng/dl) as a cut-off, sensitivity and specificity were 77.8%; (95% CI: 53–92%) and 91.3% (95% CI: 72–98%), respectively. A cut-off value of 0.08 ng/dl resulted in a specificity of 100% (95% CI: 85–100%) but low sensitivity of 11% (95% CI: 3–32%), whereas a cut-off value of 0.85 ng/dl achieved a sensitivity of 100% (95% CI: 82–100%) but lower specificity of 43.5% (95% CI: 24–64%) (Table 7).

**Table 7:** Classification of hypothyroid and NTIS dogs based on rT3 and fT4d reference intervals.

Parameter	Group	Below reference	Within reference	Above reference
rT3 (pg/ml) RI: 113–439	Hypothyroid (n = 18)	18 (100%)	0 (0%)	0 (0%)
	NTIS (n = 23)	4 (17.4%)	12 (52.2%)	7 (30.4%)
fT4 dialyzed (ng/dl) RI: 0.5–3.3	Hypothyroid (n = 18)	14 (77.8%)	4 (22.2%)	0 (0%)
	NTIS (n = 23)	2 (8.7%)	21 (91.3%)	0 (0%)

## Discussion

In our study, we successfully validated an HPLC-MS/MS method for the determination of rT3 in dogs and established a reference interval for rT3 in this species. Furthermore, we found rT3 measurement to be a useful parameter for differentiating canine hypothyroidism from NTIS showing superior diagnostic performance in sensitivity and specificity compared to fT4d for the differentiation of the two entities.

The study demonstrated the validity of rT3 measurement in dogs using HPLC-MS/MS, with good intra- and interassay accuracy and precision, a wide linearity range and good stability of serum over time. The establishment of a dog-specific reference interval for the HPLC-MS/MS method enables the clinical interpretation of future rT3 measurements in dogs. During the preparation of this manuscript, another study also reported the analytical validation of an UPLC-MS/MS method for the simultaneous determination of canine T4, T3, and rT3 and established a reference interval for rT3 in healthy dogs. Despite being generated independently in a different laboratory, the reported reference interval (107–542 pg/mL) was comparable to that established in the present study (113–439 pg/mL) [33]. This agreement supports the robustness of LC-MS/MS-based rT3 measurements and suggests good inter-laboratory comparability, particularly at the lower reference limit, which is of clinical relevance for the differentiation of hypothyroidism from NTIS.

A correlation was found between age and rT3 concentrations in the group of healthy dogs, with lower rT3 concentrations observed in older dogs (7–12 years) compared to dogs <6 years. In contrast, studies in human medicine have reported increased rT3 concentrations with advancing age. However, it has been suggested that this increase may be associated more with concurrent age-related diseases and alterations in general health status rather than aging itself [23]. These findings differ from the results of the present study, in which older healthy dogs showed lower rT3 concentrations than younger dogs. These observations in our study may be relevant for the future clinical interpretation of rT3 concentrations and particularly for dogs with NTIS in the present study (see later in the discussion), as these dogs were significantly older than dogs with hypothyroidism. However, due to the limited number of healthy dogs included, the observed age-related association of rT3 concentrations should be interpreted cautiously and verified in future studies with larger study populations.

In the second part of the study, the diagnostic performance of rT3 for the differentiation of hypothyroid dogs and dogs with NTIS was examined. We found rT3 measurement to be a useful parameter for differentiating canine hypothyroidism from NTIS showing superior diagnostic performance compared to fT4d for the differentiation of the two entities. The most promising results were observed with low rT3 levels below 52.2 pg/ml, which, in our data, were only recorded in dogs with hypothyroidism (specificity of 100%, 95% (CI: 85–100%)).

The high specificity of low rT3 values was confirmed on a conference presentation of Corsini et al. (2025) using the same rT3 assay. In their study the authors examined the diagnostic performance of rT3 to discriminate between hypothyroid dogs (n = 30) and euthyroid dogs (n = 19) with suspected hypothyroidism, that were diagnosed as euthyroid after performing gold standard diagnostics (TSH-stimulation test) [41]. As in our study the serum rT3 concentrations were significantly lower in hypothyroid dogs (median = 37 pg/ml; range 5–181 pg/ml) than in euthyroid dogs (225 pg/ml, 59–700 pg/ml). The ROC AUC value of 0.96, like the lower cut-off for rT3 of 56 pg/ml (specificity of 100%) were comparable to our data. On the other side, the cut-off for the highest sensitivity (100%) in the study was higher (181.5 pg/ml) than our cut-off (106 pg/ml) [41]. This slight discrepancy can be explained by the different study design. The dogs with hypothyroidism in our study were clear hypothyroid cases, which presumably had a more advanced disease with consequently lower levels of T4 and rT3 available. Corsini et al. were able to examine dogs with less advanced disease by diagnosing hypothyroidism using a TSH stimulation test [41]. Based on the physiology of canine hypothyroidism, it can be assumed that rT3 levels decrease as the disease progresses. If less advanced cases are included in future studies, it may be necessary to adjust the upper cut-off value to exclude hypothyroidism.

In all cases in both studies to date rT3 values below 52.2 and 56 pg/ml were diagnostic for hypothyroidism. To simplify interpretation, we recommend using a cut-off value of 50 pg/ml in clinical settings. Corsini et al. were also able to demonstrate a substantial (K = 0.8, 95% CI: 0.58–1.0) correlation between rT3 values and stimulated tT4 values in the TSH stimulation test, which underscored the diagnostic significance of rT3 [41]. From a clinical perspective, this suggests that rT3 could serve as a valuable adjunctive parameter, particularly in cases with inconclusive tT4 or TSH results for the differentiation of hypothyroidism and NTIS. Incorporating rT3 into the diagnostic work-up may therefore improve diagnostic accuracy and reduce the risk of misclassification.

As another part of our study, we compared the clinical performance of rT3 and fT4d. Importantly, rT3 demonstrated slightly superior diagnostic performance compared to fT4d for differentiating hypothyroidism from NTIS, as reflected by the AUC of the ROC analysis. In our study, only 77.8% of hypothyroid dogs showed fT4d concentrations below the lower reference limit, whereas rT3 concentrations were below the lower reference limit in all hypothyroid dogs resulting in better sensitivity for rT3. This is in accordance with other studies showing moderate sensitivity of 71–90% [17] of fT4d for diagnosing hypothyroidism [20]. The optimal cut-off for best specificity of both parameters (52.2 pg/ml rT3 and 0.08 ng/ml fT4d) is combined with still good sensitivity for rT3 (82.6%) and a low sensitivity in fT4d (11%). The exact pathophysiological mechanisms underlying the reduction in rT3 levels in dogs with hypothyroidism and NTIS are currently unknown. On the other hand, a number of studies have investigated the diagnostic performance of fT4d in dogs with NTIS and hypothyroidism. In contrast to data of older studies that reported fT4d to be less influenced by NTIS [3] the study of Bennaim et al. showed values of fT4d under the reference interval in 57.1% in dogs with NTIS [19]. This finding is in contrast to our data, where only 2 out of 23 (8.7%) dogs with NTIS showed low fT4d values.

Possible explanations for the slightly different discriminatory performance of rT3 and fT4d may theoretically be explained by physiological differences between these parameters. While fT4d primarily reflects the circulating free fraction of thyroxine, rT3 may more directly reflect peripheral thyroid hormone metabolism. In human medicine, NTIS is associated with altered peripheral deiodination, including reduced conversion of T4 to biologically active T3 and increased formation of rT3 [26,42]. Consequently, rT3 may more specifically reflect metabolic adaptations associated with systemic illness, whereas fT4 concentrations can be influenced by multiple factors including assay interference, disease severity, transport protein alterations, and residual thyroid function. Whether identical mechanisms occur in dogs remains to be elucidated.

In human medicine, patients with NTIS frequently show elevated rT3 concentrations, with up to 75% of hospitalized patients exhibiting increased levels [25,27]. In contrast, in our study a broad range of rT3 values in dogs with NTIS (52.2 to 700 pg/ml) was identified with only 7/23 dogs (30.4%) showing rT3 concentrations above the upper range of the reference limit. Most of the dogs (52.2%) had rT3 levels within the reference interval or even below the reference interval (2/23; 8.7%). One possible reason for this broad range of results in our study can be explained by very different underlying acute and chronic diseases. A study in men by Fliers (2021) suggests that varying regulation of T4 transporters, deiodinases, and T4 receptors in acute and chronic diseases, could explain why rT3 values are often lower in acute cases compared to chronic conditions. Furthermore, reduced nutritional intake during acute illness phases may influence thyroid hormone metabolism, leading to higher conversion of T4 to rT3 providing a beneficial adjustment during acute illness [42].

Among the dogs with NTIS in our study, there was considerable variability in underlying conditions, and in some cases it was not always clear whether the condition was acute or chronic. The two dogs with rT3 levels below the reference interval presented with chronic gastrointestinal haemorrhage and pyelonephritis, in which the duration of the disease could not be assessed. The 7 dogs with high rT3 levels tended to present with acute and, above all, very severe conditions. Several factors may explain the observed differences between dogs and humans. Species-specific variations in thyroid hormone metabolism, particularly in deiodinase activity, may play a role. In humans with NTIS, reduced type 1 deiodinase activity and increased type 3 deiodinase activity contribute to decreased T3 and increased rT3 concentrations. Whether these mechanisms occur to the same extent in dogs remains unclear [23,40,43,44].

A further consideration concern the age distribution of the study population. Dogs in our study with NTIS were significantly older compared to dogs with hypothyroidism. Since older dogs are more likely to suffer from severe underlying diseases and hypothyroidism is known to be a disease of middle-aged dogs, the significant age difference can be explained by the pathogenesis of the diseases. Within the group of healthy dogs, an age-related decline in median rT3 values was observed. This increases the statistical significance of high rT3 values in old NTIS dogs compared to low rT3 values in middle-aged hypothyroid dogs. An age-related false increase of rT3 in dogs with NTIS therefore seems unlikely.

Based on our findings, further investigations should focus on the evaluating of rT3 in dogs with varying disease severities, different underlying conditions associated with NTIS, and in cases of subclinical hypothyroidism, where diagnostic differentiation remains particularly challenging. In addition, combined assessment of rT3 and tT3 may provide further insight into thyroid hormone metabolism and should be explored in future studies.

Two of 23 (8.7%) patients with NTIS had elevated TSH concentrations in our study. This is in accordance with the literature, where in one study, 21.7% of NTIS dogs showed elevated TSH [5] while another study reported that 4% and 12% of canine NTIS patients had TSH levels above the reference interval after 2 and 4 weeks of recovery, respectively [4,38,42,44–46]. Our data confirm those diagnostic difficulties of differentiating between hypothyroidism and NTIS when relying on the interpretation of TSH and tT4 alone.

One important limitation of this study is the small sample size of dogs with hypothyroidism and NTIS leading to statistical variability, as can be seen from the wide confidence intervals of the calculated sensitivities and specificities. Nevertheless, the high significance of rT3 levels between dogs with hypothyroidism and NTIS demonstrates the fundamental validity of rT3 measurement for distinguishing between the two conditions. Follow-up studies involving larger sample sizes may therefore necessitate an adjustment of cut-off values for distinguishing between the conditions and a revision of sensitivities and specificities. The data we have collected should therefore be viewed as preliminary and should be updated with larger case numbers.

Another limitation of the present study is the absence of a definitive gold standard for the diagnosis of hypothyroidism and NTIS such as thyroid scintigraphy or TSH stimulation testing [7,9,11]. Instead, a composite reference standard based on clinical findings and laboratory parameters in combination with improvement following levothyroxine therapy (hypothyroid dogs) was applied, which cannot definitively rule out the occurrence of individual misclassifications. However, this approach reflects routine clinical practice, where diagnosis is typically based on a combination of clinical presentation and laboratory results.

The selection criteria for dogs with NTIS may also represent a limiting factor in this study. Of the 47 dogs initially classified as non-thyroidal diseases, only 23 exhibited decreased T4 concentrations. Dogs with mild non-thyroidal diseases did not show reduced T4 levels and were therefore not included, as our aim was to focus on clear cases with low T4 values to ensure more distinct group allocation and stronger analytical power. This selection approach, however, may have excluded dogs with less severe systemic illness. Kantrowitz et al. (2001) similarly demonstrated that tT4 concentrations decrease more markedly in dogs with severe underlying disease compared to those with milder conditions [3].

In conclusion, this study demonstrates that serum rT3 measurement using a validated HPLC-MS/MS method is a promising diagnostic tool in canine thyroid diagnostics. rT3 showed excellent analytical performance and enabled the establishment of a dog-specific reference interval, forming the basis for clinical interpretation. Most importantly, rT3 proved to be highly effective in differentiating hypothyroidism from NTIS and outperformed fT4d in this regard, particularly due to its high specificity at low concentrations.

Future studies with larger and more heterogeneous cohorts are warranted to validate the proposed cut-off values and further elucidate the role of rT3 in different stages and severities of disease. In addition, combined assessment of rT3 with other thyroid hormones may further improve diagnostic accuracy in complex clinical cases. Overall, rT3 represents a valuable adjunct parameter that has the potential to significantly improve the differentiation of hypothyroidism and NTIS in dogs in clinical practice.

## Supporting information

S1 Table Baseline characteristics of healthy dogs. (PDF)

S2 Table Baseline and thyroid hormone parameters in hypothyroid dogs. (PDF)

S3 Table Baseline and clinical characteristics of dogs with NTIS. FI – female intact, FS – female spayed, MI – male intact, MN – male neutered, RI – Reference interval, n.d. – not determined.
(PDF)
