# Supplementary material for: Serum reverse triiodothyronine in dogs with hypothyroidism, nonthyroidal illness syndrome and healthy dogs
Source: PLoS One. 2026 Oct 5;21(10):e0357106. doi: 10.1371/journal.pone.0357106 (PMC13637879; doi:10.1371/journal.pone.0357106)
Supplement: Supplementary file 1 [file pone.0357106.s001.pdf]

S1 Table. Baseline characteristics of healthy dogs.

	Signalement						
**Patient ID**	**Breed**	**Age (years)**	**Sex**	**Weight (kg)**	**tT4 (ng/dl) RI: 1.5-4.5**	**TSH (ng/ml) RI: <0.59**	**rT3 (pg/ml) RI: 113-439**
01	Biewer Yorkshire Terrier	8	FI	2.3	3.21	0.10	251
02	Mixed breed	5	MI	22	2.15	0.16	334
03	Wirehaired Viszla	2	MI	34	1.81	0.23	318
04	Wirehaired Viszla	6	FS	22	2.5	0.15	218
05	Beagle	12	FS	16	1.99	0.19	111
06	Mixed breed	8	MN	22	2.39	0.16	277
07	Mixed breed	5	MN	28	2.02	0.12	213
08	Malonois	3	FS	25	2.26	0.12	214
09	Australian Shepherd	5	FS	16	2.45	0.07	281
10	Cavalier King Charles Spaniel	9	FS	6	2.61	0.20	231
11	Mixed breed	2	FS	13	2.97	0.09	246
12	Mixed breed	3	MN	16	1.89	0.10	154
13	Weimaraner	3	FI	30	2.50	0.07	296
14	German Hunting Terrier	2	FS	10	2.80	0.10	250
15	German Longhaired Pointer	5	MI	33	2.16	0.16	272
16	Mixed breed	9	FS	27	1.56	0.14	184
17	Mixed breed	6	FS	10	2.26	0.23	258
18	Shorthaired Vizsla	9	MN	28	2.45	0.12	246
19	Dachshund	4	FI	9.5	2.77	0.27	327
20	Labrador Retriever	5	MN	30.3	3.10	0.14	347
21	Mixed breed	6	MI	30	3.52	0.10	143
22	Bavarian Mountain Hound	2	MN	23	2.81	0.11	392
23	Weimaraner	2	FI	27.5	2.33	0.11	350
24	Lagotto Romagnolo	2	FI	10	3.43	0.15	616
25	Cockerpoo	5	MI	10	1.81	0.17	260
26	White Swiss Shepherd Dog	4	MN	42	1.67	0.11	191
27	Australian Shepherd	2	MI	25	1.98	0.16	327
28	Labrador Retriever	2	FI	24.5	3.68	0.09	817
29	German Shorthaired Pointer	7	MN	27	1.82	0.11	131
30	Mixed breed	4	MI	45	2.50	0.20	349
31	Mixed breed	2	MI	19	2.54	0.11	408
32	Weimaraner	3	FI	23	3.35	0.17	310
33	Labrador Retriever	5	FS	22	2.41	0.14	440
34	Wirehaired Viszla	1	MI	24	2.01	<0.03	316
35	Dalmatian	12	FS	22	1.64	0.18	233
36	Dalmatian	5	FS	24	2.30	0.30	397
37	Mixed breed	10	FI	30	1.78	0.25	184
38	Mixed breed	8	FS	12	2.31	0.12	288
39	White Swiss Shepherd Dog	5	FI	28	1.78	0.30	207
40	Australian Shepherd	10	FS	21	2.75	0.12	321
41	Malinois	2	MI	27	2.58	0.11	586
42	Mixed breed	5	MN	10	1.44	0.12	425
43	Mixed breed	7	MN	24	1.70	0.46	190
44	German Shepherd	4	MI	34	1.54	0.18	245
45	Mixed breed	5	MN	6.3	1.85	0.12	328
46	Mixed breed	5	FI	19.6	1.47	0.33	337
47	Mixed breed	5	MI	18	1.59	0.18	346
