# Supplementary material for: Serum reverse triiodothyronine in dogs with hypothyroidism, nonthyroidal illness syndrome and healthy dogs
Source: PLoS One. 2026 Oct 5;21(10):e0357106. doi: 10.1371/journal.pone.0357106 (PMC13637879; doi:10.1371/journal.pone.0357106)
Supplement: Supplementary file 2 [file pone.0357106.s002.pdf]

S2 Table. Baseline and thyroid hormone parameters in hypothyroid dogs.

	Signalement				Symptoms															
**Patient ID**	**Breed**	**Age (years)**	**Sex**	**Weight (kg)**	**Lethargy**	**Weight gain**	**Cold intolerance**	**Alopecia**	**Seborrhea**	**Pruritus**	**Pyoderma**	**Ear infection**	**tT4 (ng/dl) RI: 1.5-4.5**	**TSH (ng/ml) RI: < 0.59**	**fT4 dialyzed (ng/dl) RI: 0.47-3.26**	**tT3 (ng/ml) RI: 0.8-2.1**	**T3AA % RI: 0-10**	**T4AA % RI: 0-20**	**TgAA % RI: <35**	**rT3 (pg/ml) RI: 113-439**
01	Flat Coated Retriever	10	MI	33	x							x	0.70	1.75	0.78	0.98	4	11	5	106
02	Dalmatian	5	MI	28									0.46	8.21	0.39	0.59	7	10	25	98,8
03	Labrador Retriever	7	FI	35				x	x	x		x	<0.12	1.23	0.08	0.32	9	15	8	2.5
04	Mixed breed	10	MI	30	x	x		x	x	x		x	0.13	0.67	0.08	0.39	5	10	4	2.5
05	Perro de Agua Español	6	MN	20	x	x		x					0.20	6.33	0.16	0.26	23	14	83	2.5
06	German Shepherd	5	MI	35	x		x						0.13	2.62	0.39	0.46	6	15	12	2.5
07	Miniature Bull Terrier	4	FS	11	x		x	x					<0.12	1.82	0.16	0.52	5	13	4	2.5
08	Havanese	8	FS	5			x	x	x	x	x		<0.12	0.99	0	0.26	14	18	87	2.5
09	Mixed breed	4	MN	4	x	x	x						<0.01	1.76	0.08	0.52	12	17	139	16,8
10	Mixed breed	3	MN	-	x	x							<0.12	2.03	0.08	0	65	24	138	2.5
11	Mixed breed	8	FS	25			x		x	x			0.28	5.69	0.47	0.46	7	13	4	2.5
12	Havanese	9	MI	9.8	x	x						x	<0.12	1.30	0.23	0	20	13	14	2.5
13	Mixed breed	6	MI	30	x	x		x	x				<0.12	4.80	0	0	4	15	9	2.5
14	Mixed breed	6	FS	27.5		x			x				<0.12	6.15	0.08	0	90	21	166	2.5
15	Mixed breed	2	FS	18.3	x	x				x			1.34	6.76	0.54	0	33	27	201	65,1
16	Mixed breed	4	MN	29.6	x	x							0.12	6.34	0.54	0.39	6	9	20	37,6
17	Mixed breed	3	MN	59	x	x							<0.5	2.62	0.08	0.07	15	13	35	2.5
18	Cane Corso	4	MI	46	x		x	x	x				<0.12	1.33	0.08	0	83	10	130	2.5
