# Supplementary material for: Serum reverse triiodothyronine in dogs with hypothyroidism, nonthyroidal illness syndrome and healthy dogs
Source: PLoS One. 2026 Oct 5;21(10):e0357106. doi: 10.1371/journal.pone.0357106 (PMC13637879; doi:10.1371/journal.pone.0357106)
Supplement: Supplementary file 3 [file pone.0357106.s003.pdf]

S3 Table. Baseline and clinical characteristics of dogs with NTIS.

	Signalement												
**Patient ID**	**Breed**	**Age (years)**	**Sex**	**Weight (kg)**	**Disease**	**tT4 (ng/dl) RI: 1.5-4.5**	**TSH (ng/ml) RI: < 0.59**	**fT4 dialyzed (ng/dl) RI: 0.47-3.26**	**tT3 (ng/ml) RI: 0.8-2.1**	**T3AA % RI: 0 - 10**	**T4AA % RI: 0 - 20**	**TgAA % RI: <35**	**rT3 (pg/ml) RI: 113-439**
01	Russian Terrier	12	MI	33.3	multimorbid: peripheral neuropathy, recurrent aspiration pneumonia, diabetes mellitus	0.20	0.22	0.47	0.07	3	88	1	235
02	Hovawart	2	MI	41	septic abdomen due to an intrahepatic foreign body	0.27	0.35	0.78	0.46	5	11	10	72
03	Mixed breed	8	FI	23	xylitol intoxication	0.31	<0.03	0.47	0.20	3	5	3	52,2
04	Labrador Retriever	12	MN	40	pericardial effusion, ascites, high-grade thrombocytopenia	0.29	0.14	1.71	0.65	5	6	6	200
05	Mixed breed	6	MN	11.6	chronic leishmaniasis	0.86	0.11	1.09	0.39	3	7	13	228
06	Mixed breed	15	FI	21.4	second-degree atrioventricular block	1.16	0.41	0.54	0.65	3	11	4	635
07	Pug	8	FI	6.5	pyelonephritis	0.22	0.75	0.70	0.33	6	6	9	162
08	Rhodesian Ridgeback	9	FS	29	pyelonephritis	1.25	0.60	1.01	0.39	4	4	3	99
09	Dachshund	13	MI	13.8	chronic gastrointestinal bleeding of unknown etiology	<0.12	0.18	0.85	0.59	4	7	2	92
10	Australian Shepherd	10	MI	26	increasing weakness, polyorie/polydipsia and alopecia - no follow up	0.60	0.03	0.70	0.26	5	9	2	152
11	Mixed breed	14	MN	30	suspicion of Cushing, additional acute blindness - further examination by another vet	0.72	0.19	1.09	0.2	4	4	3	229
12	Golden Doodle	7	MN	29.5	babesiosis with secondary acute kidney injury	0.13	0.23	0.47	0	1	3	4	251
13	Maltese	3	FI	6.6	septic abdomen due to intestinal perforation caused by foreign body	<0.12	0.12	1.63	0	4	4	4	455
14	Shetland Sheep Dog	12	FS	8.4	transitional cell carcinoma of the bladder, hepatocellular carcinoma	<0.12	0.53	0.08	0	0	7	1	175
15	Golden Retriever	14	FS	26	megaesophagus, aspiration pneumonia, exitus letalis	<0.12	<0.03	0.08	0	5	7	10	700
16	Mixed breed	1	FI	34	pyometra, abscess/phlegmon on the left lateral side of the larynx	0.43	<0.03	0.62	0.26	3	9		579
17	Golden Retriever	11	MI	42.5	high grade mast cell tumor with metastasis	1.06	0.06	0.78	0.72	5	11	6	148
18	Mixed breed	15	FS	17.5	otitis externa, dermatitis, hypotrichosis, Cushing disease	0.98	0.10	0.70	0.65	6	9	4	269
19	Labrador Retriever	3	MI	22.5	pancreatitis, secondary gastropathy, diabetes mellitus, pyogranulomatous inflammation in the inguinal region, exocrine pancreatic insufficiency, pyoderma	1.11	0.11	2.72	0.13	0	2	6	615
20	Mixed breed	11	FI	22.6	protein loosing eneteropathy, Diarrhoea, Hypalbuminaemia	1.27	0.17	1.09	0.33	4	9	2	120
21	Mixed breed	7	MN	35	car accident, pneumothorax, rib fx 5th + 6th rib on the right	0.96	0.18	1.24	0.52	5	8	2	569
22	Mixed breed	7	MN	34.1	vestibular syndrome and facial paresis on the right, mainly inflammatory, weight loss	1.09	<0.03	0.78	0.59	3	8	5	273
23	Dachshund	4	FS	8.4	ANI post section porro due to an avital fetus	1.11	0.15	1.63	0.33	6	11	4	555
