# Supplementary material for: Serum reverse triiodothyronine in dogs with hypothyroidism, nonthyroidal illness syndrome and healthy dogs
Source: PLoS One. 2026 Oct 5;21(10):e0357106. doi: 10.1371/journal.pone.0357106 (PMC13637879; doi:10.1371/journal.pone.0357106)
Supplement: Supplementary file 4 [file pone.0357106.s004.docx]

**General comments**

I would like to thank the authors for sharing these interesting and original data and compliment them on their work. The manuscript presents relevant data on the use of rT3, including validation of a new assay in dogs, definition of a reference interval, and a diagnostic accuracy study for hypothyroidism. However, in my opinion, many issues remain to be clarified, and the manuscript should be thoroughly revised.

Materials and Methods and the Results sections must be revised (see specific comments) to better adhere to the STARD guidelines for reporting diagnostic accuracy studies (https://www.equator-network.org/reporting-guidelines/stard/).

In the Abstract and Results sections, I strongly suggest adding effect estimates (e.g., median differences) with 95% confidence intervals for both statistically significant and non-significant results. This approach gives more precise information to the reader, allowing for a better interpretation of the results beyond the P value.

I suggest re-assessing the Discussion to better focus on the many results reported in the study, rather than reporting a general discussion of the pathophysiological mechanisms, which seems beyond the scope of the manuscript. Furthermore, some results require a more detailed discussion.

I also suggest reviewing the most recent literature, as many of the citations are outdated. Indeed, reported citations are appropriate, but integration with the most recent publications is desirable.

Regarding the literature search, I suggest defining how it was conducted. Rather than simply stating ‘there are no studies’ or ‘based on the author's knowledge’, it is preferable to add a line that defines how the search was performed (https://onlinelibrary.wiley.com/doi/10.1111/vru.12748).

For example, instead of lines 73-74 ‘Comparative studies on rT3 measurement in dogs are not currently available’, the authors can state ‘The following databases (XX, XX and XX) have been searched with the following keywords XX XX on [date, eg 01/01/2020], and no other reports of XX have been found’ or a similar formulation. This also allows the authors to modify the discussion. Whenever referencing in the Discussion (or any other section) to this, you can use statements like "Based on the literature search, this is the first time ..." or similar (see lines 254 and 273).

**Specific comments**

Abstract

Line 29: Median value is 2.5, while the lower limit of the range is <5. I am not sure if this is the correct way to report these results. If you define values <5 as equal to 2.5 for statistical reasons (line 162-163), you should probably report the lower limit of the range equal to 2.5. Please check and, if the same issue occurs, report it in the results section.

Line 32-33: Please add the confidence interval for the sensitivity and specificity.

Introduction

Line 46: When citing multiple studies, you should report the results of all these studies. There are different possible approaches. First, you can cite all the results (e.g., 31%, xx %, and xx %); second, you can just report the range (e.g., 31% to xx%, or xx% to 31%), as you did in line 65; finally, if you choose this percentage because it is the highest one reported, clearly state this (e.g., in up to 31%). Of note, you reported a range of prevalence in the abstract (line 16).

Line 47: I suggest revising this statement. Even though a clear definition of NTIS is difficult to derive solely from T4 measurements (which is the definition of NTIS?), it is generally accepted that low T4 is a criterion for defining the occurrence of NTIS in veterinary medicine, since we do not routinely measure T3. If not, all dogs with any disease should be considered as affected by NTIS. In this context, it is more appropriate to use the definition ‘non-thyroidal illness’ (without syndrome) or simply ‘concurrent diseases’, with the second one probably avoiding some confusion between ‘non-thyroidal illness’ and ‘non-thyroidal illness syndrome’.

Line 48: ‘Stimulation’ instead of ‘response’. Also, I guess the definition of "non-invasive" could be called into question. Indeed, thyroid scintigraphy is certainly demanding, but not necessarily invasive (see comments on line 53). I suggest using ‘gold standard among the blood tests’, or something similar.

Line 48-51: Please check the most recent literature: https://onlinelibrary.wiley.com/doi/10.1111/jsap.13290

Line 53: Is it always the case? It probably depends on the protocol used, but I guess that thyroid scintigraphy can also be performed under sedation.

Line 61: see comment on line 46 about the use of percentage extrapolated from the literature.

Line 58-63: Free T4-ED is not even mentioned among possible diagnostic tests. Since it is usually considered the most accurate test among the non-dynamic tests, I guess it is worth mentioning. It is even more important, given that the authors compared rT3 and fT4-ED in their study.

Line 66: Since rT3 is the core of the study, a brief description of rT3 is due before describing its use in humans and veterinary medicine. Furthermore, is it routinely used in humans?

I would consider scaling back the assumption about the usefulness of rT3 in humans: ‘has been suggested as a useful test to…’ instead of ‘is useful’?

Line 69-73: Please specify if total T3 (tT3) or free T3 (fT3).

Line 70: ‘Lower’ instead of ‘low’

Line 75: I suggest adding the validation of the assay as an aim of the study. It is an important result reported by the authors, but not adequately highlighted. Also, it seems that another aim of this study could be to compare rT3 and fT4-ED (see comment on lines 58-63).

Line 78: The study's aims should be followed by the author's hypothesis, at least for the comparative part of the study (e.g., We hypothesized that rT3 accurately…).

M&M:

The study design is not clearly stated. ‘Prospective’ is not a study design, simply describing how dogs were included time-wise. In my opinion, the study has two distinct parts that should be described appropriately without overlap; the authors did so in the results section but not in the MMs section. The first part involves assay validation and the definition of a reference interval; the second part of the study is a diagnostic accuracy study assessing the performance of rT3. I recommend consulting STARD guidelines for reporting diagnostic accuracy studies, https://www.equator-network.org/reporting-guidelines/stard/. The M&M section should be revised accordingly.

The main problem here is the definition of the reference standard: there is no specific test against which to compare rT3, but the proposed composite reference standard is somewhat acceptable from a clinical perspective. Still, this limitation should be clearly stated in the discussion, as you partially did in lines 336-340.

Line 86-90: The original population from which dogs were included should be better defined, as well as the process leading to the inclusion of dogs in the groups. For example, what were the criteria used to consider a dog a potentially eligible participant (e.g., all dogs presented in the selected period in which a thyroid panel was performed)? Have you decided a priori how many dogs to include in each group, or do you include all dogs that meet the appropriate criteria?

Related to this, it seems that healthy dogs were included differently from the dogs used in the diagnostic accuracy study.

Line 92: Pruritus is not a clinical sign of hypothyroidism. Indeed, it can be present if there is concurrent pyoderma, but it should not be included among clinical signs suggesting hypothyroidism.

Line 97-98: What does ‘moderate to severe’ underlying disease mean? Which criteria were used to define a disease as moderate to severe?

Furthermore, how was hypothyroidism ruled out in NTIS dogs showing low tT4 and high TSH?

Line 106: Whenever drug administration is used as an exclusion criterion, the time should be clearly stated. Was only ongoing treatment considered, or also previous treatment? In the second case, for how long had the drug to be discontinued before inclusion?

Line 108: Were TKIs included in the list of drugs leading to exclusion from the study? If not, can you please explain why?

Line 121: The commercial name of the profile is irrelevant; I would simply report the parameter measured in each dog (e.g, ‘complete blood count, serum chemistry profile including……, and total T4 and TSH were measured by Antech Lab’)

Line 130: Please specify the assays used to measure tT4 and TSH. I guess the lab used Siemens Immulite canine T4 and canine TSH, but it should be clearly stated. Also, specify if the reported intervals were calculated by the Lab.

Line 133-141: I would consider reporting this section as the results of the validation of the assay, rather than MM. These data are relevant, given that this assay was not previously validated in dogs (see line 75).

Line 143-151: It is not clear to me why all these parameters, except for FT-ED, were measured in both groups. Indeed, the results about tT3, T4AA, T3AA, and TgAA were not analyzed (not even descriptively) nor discussed. As it is, they seem quite out of context. Nonetheless, some of this data could probably be analyzed in an interesting way. For example, based on what is described in lines 66-69, has the author considered the possibility of analyzing the T3 to rT3 ratio in these dogs?

It clearly was not planned a priori, but the authors could conduct it as a post hoc analysis and appropriately report it in the Statistical Analysis section.

Line 154: Why were the data expressed as median and range? Was it due to non-normal distribution? How was the distribution of the data assessed? Furthermore, please be aware that range is not a measure of dispersion for non-normally distributed data; these should be described using other measures (e.g., the interquartile range). For this reason, IQR provides a better assessment of the variability of a dataset compared to range.

Line 156-157: I suggest to clearly state which method was used here, and not in the results (line 194-195). E.g., ‘…was calculated using a robust untransformed…based on the…’

Line 159: This sentence is not clear. The authors must describe here in the MM the analysis performed. E.g., ‘In healthy dogs, rT3 values were compared between young (healthy dogs <6 years) and old dogs (healthy dogs >/=7 years) using…’. Also, if the authors want to explore the correlation between age and rT3 values, it could be interesting to perform a correlation analysis (e.g. Spearman’s correlation).

Line 161: The ROC curve analysis was performed for both rT3 and FT4-ED, but the latter is not reported here. Please add it.

Line 164: See also the general comment of estimates of effects and confidence interval. Please add this part in the statistical section.

Results:

Line 167-168: I strongly suggest adding a flowchart at the beginning of this section, to highlight how the population in the diagnostic accuracy part of the study was created, as suggested by the STARD guidelines https://www.equator-network.org/wp-content/uploads/2015/03/STARD-2015-flow-diagram.pdf. Also, considering dogs were included from many centers, the number of dogs included in each center (Haar, University and lab-referred) should be clarified.

Line 176-181: Please avoid the use of ‘tends to’ or reporting population as younger or older if statistical significance is not achieved. To make these comparisons clearer, I suggest summarizing data about sex, age and body weight in a Table, describing only the comparisons with median difference and confidence intervals in the text. E.g., ‘No age difference was observed between hypo and healthy dogs (MD = xxx, 95% CI xx-xx, p= xxx) nor between NTIS and hypo dogs (MD = xxx, 95% CI xx-xx, p= xxx), while NTIS were older compared to healthy dogs (MD = xxx, 95% CI xx-xx, p= xxx),’. This way, the reader has all data to critically analyze the results reported, without the use of speculative terms such as ‘tends to’.

The same suggestions apply to all the Results section.

Figure 1: See previous comment. I suggest to graphically underline the significant differences between NTIS and healthy dogs.

Line 188: I would put the results of the validation here, along with the ones regarding the reference interval, in a dedicated paragraph. E.g., ‘Validation of rT3 assay and reference interval in healthy dogs.’

Table 1, 2, or 3 and lines 200-209: I suggest moving these three tables in the Supplementals. Even if the possibility to look at single dog is interesting, I do not believe it is really required in the main texts. Rather, I would summarize the descriptive analysis (median, IQR, range) regarding T4, TSH, rT3, and FT4 in the three groups in a single table, reporting the results of the comparisons in the text (see previous comments).

Furthermore, it could be interesting to know how many dogs (percentage) in hypo and NTIS group showed rT3 and fT4-ED values below (which means a positive test), within, or above (which means a negative test) the reference interval. In a diagnostic accuracy study, this is more valuable than a comparison between median values of healthy dogs and hypo/NTIS dogs: to extremize the concept, median values of dogs in hypo/NTIS groups could hypothetically be lower compared to healthy ones, but still, most of the single value of each dog could remain within the reference interval.

Figure 2: I suggest to graphically show the reference interval, to emphasize the distribution of the rT3 below, within or above the RI in the three groups.

Line 227: This sentence should be in the statistical analysis section.

Line 228-232 and 238-242: Please add the confidence interval for sensitivity and specificity values.

Figure 3 and 4: I would consider joining these two figures, to better emphasize the comparison between the ROC curves of the different tests.

Discussion:

Line 250-253: The incipit of the discussion usually summarizes the most relevant results of the study. In my opinion, I would add here the validation of the assay and the better performance of the rT3 compared with fT4-ED.

Line 254-255: The authors stated there are no comparative studies, but later they discuss the results of Corsini et al. 2025 (line 294-315). I agree with the authors that there are no studies published on peer-reviewed journals (provided their literature search confirm this assumption – see general comments), since Corsini et al. is a conference presentation and those results are unpublished to date. However, this discrepancy is not adequately discussed. I would probably consider at least underlining the nature of the Corsini at al. study in the manuscript.

Line 256-274: This entire paragraph is out of focus and should be removed from the discussion. This is merely a pathophysiological discussion about rT3 and NTIS, which is not related to the results reported by the authors. Indeed, the discussion must focus on the results presented in the study. This section could be moved, in a shorter and summarized version, in the Introduction to give the reader some information on what rT3 is and why it could be useful.

Line 276-277: see previous comments

Line 279-281: These are results, that should not be repeated in the discussion. Rather, discuss the implication of these results, if considered relevant.

Line 281-283: That’s true, but is this clinically relevant? We do not use any of this test to discriminate healthy and hypothyroid dogs, but rather to discriminate dogs with clinical signs (or laboratory abnormalities) due to hypothyroidism or due to NTIS.

Line 284-285: Please, go into details. For example, you should comment on the discrepancy in the percentage of dogs with NTIS with high rT3 (which must be added in the results, see previous comments) compared to humans. Which are the possible explanations? Maybe assessing tT3 and rT3 (see previous comments) can give you some idea.

Line 290-293: This seems to me the most interesting and clinically relevant result of the present study. I would highlight it and discuss it properly.

Line 317: ‘is’ rather than ‘could be explained’

Line 328-329: Please check the literature: https://academic.oup.com/jvim/article/38/3/1345/8456062

Line 332: It may be true, but it is not explained why the sample size limits the interpretation of the Results. For example, smaller samples lead to decreased statistical power; of note, this study has no power analysis, and this is a limitation worthy to remember. The (hypothetical) low statistical power increase the risk of type II error (failing to detect a difference that exist); however, does not seem the case considering the authors were able to demonstrate their hypothesis. Another possible limitation related to the low statistical power, is that the confidence intervals could be very wide; however, since now confidence intervals are not reported for most of the results, I am not able to comment on this (and so is the hypothetical reader).

Line 336-339: See previous comments, reported here - The main problem here is the definition of the reference standard: there is no specific test against which to compare rT3, but the proposed composite reference standard is somewhat acceptable from a clinical perspective.

Line 340-341: This is a result. The fact that rT3 was rechecked after treatment or after resolution of the concomitant diseases in a subset of dogs should be clearly described in the MM section/Study design, and these lines moved from the Discussion to the Results. Then, you can refer to the result to support you argumentation here.

Line 357-358: This is well discussed, but it could be even better to have a correlation analysis (see previous comments).

Line 361-364: Same comment as lines 250-253

Line 364-368: I would remove these lines. Generically stating the need of further studies is of no value. Rather, the authors could focus on the indication of performing more studies in dogs with different conditions or subclinical hypothyroidism, if they believe those are the settings that should be further investigated based on their results.

References

Line 472: This citation should be revised (https://doi.org/10.1093/jvimsj/aalaf018). Please check the guidelines for citing conference abstracts.
