# Supplementary material for: Serum reverse triiodothyronine in dogs with hypothyroidism, nonthyroidal illness syndrome and healthy dogs
Source: PLoS One. 2026 Oct 5;21(10):e0357106. doi: 10.1371/journal.pone.0357106 (PMC13637879; doi:10.1371/journal.pone.0357106)
Supplement: Supplementary file 5 [file pone.0357106.s005.docx]

**Response to the Academic Editor**

Dear Editor,

Thank you for all the efforts related with the handling of our manuscript and for giving us the opportunity to revise our study. Based on the excellent comments provided by you and the reviewers, we have decided to fundamentally revise the manuscript, or rather, to revise large portions of it. In the following, we explain in greater detail how we dealt with each of your comments while revising the manuscript. We first repeat your comments (in italics) and then provide our answers. Further below, you find our point-by-point reply to each of the reviewers’ comments.

**Editor:**

*1. Please ensure that your manuscript meets PLOS ONE's style requirements, including those for file naming. The PLOS ONE style templates can be found at*

[*https://journals.plos.org/plosone/s/file?id=wjVg/PLOSOne_formatting_sample_main_body.pdf*](https://journals.plos.org/plosone/s/file?id=wjVg/PLOSOne_formatting_sample_main_body.pdf) *and*

[*https://journals.plos.org/plosone/s/file?id=ba62/PLOSOne_formatting_sample_title_authors_affiliations.pdf*](https://track.editorialmanager.com/CL0/https:%2F%2Fjournals.plos.org%2Fplosone%2Fs%2Ffile%3Fid=ba62%2FPLOSOne_formatting_sample_title_authors_affiliations.pdf/1/010f019d1e310623-ad2f698f-517f-4e0a-bba9-86dd07c06e8b-000000/7AGhtkFURvoyoKbrbDYygxhgefeW6w0Z4cigCaK02ZE=255)

**Answer:** We carefully went through the manuscript and complied as requested.

**Editor:**

*2. Thank you for stating the following in the Acknowledgments Section of your manuscript:*

*"We thank the laboratory teams at Antech Lab Germany and SYNLAB MVZ Munich GbR for their technical support. We also gratefully acknowledge the DVGD (Deutsche Veterinärmedizinische Gesellschaft für Dermatologie) for financial support through a research grant."*

*We note that you have provided additional information within the Acknowledgements Section that is not currently declared in your Funding Statement. Please note that funding information should not appear in the Acknowledgments section or other areas of your manuscript. We will only publish funding information present in the Funding Statement section of the online submission form.*

*Please remove any funding-related text from the manuscript and let us know how you would like to update your Funding Statement. Currently, your Funding Statement reads as follows:*

*"This study was financially supported (JR) by a research grant from the DGVD – Deutsche Gesellschaft für Veterinärdermatologie (German Veterinary Dermatology Society), which is gratefully acknowledged.*

*URL:* [*https://www.dgvd.org/*](https://www.dgvd.org/)

*Grant Number: 01/2022"*

*Please include your amended statements within your cover letter; we will change the online submission form on your behalf.*

**Answer:** We have removed the statement in question

The Role of Funder statement has been amended accordingly. Financial support was provided by the DGVD – Deutsche Gesellschaft für Veterinärdermatologie (German Veterinary Dermatology Society). Additional laboratory-associated costs and scientific support were provided by Antech Diagnostics Germany. Representatives of Antech Diagnostics Germany contributed to study design, data analysis, statistical evaluation, and critical revision of the manuscript..

**Editor:**

*3. Thank you for stating the following financial disclosure:*

*"This study was financially supported (JR) by a research grant from the DGVD – Deutsche Gesellschaft für Veterinärdermatologie (German Veterinary Dermatology Society), which is gratefully acknowledged.*

*URL:* [*https://www.dgvd.org/*](https://www.dgvd.org/)

*Grant Number: 01/2022"*

*Please state what role the funders took in the study.  If the funders had no role, please state: "The funders had no role in study design, data collection and analysis, decision to publish, or preparation of the manuscript."*

*If this statement is not correct you must amend it as needed.*

*Please include this amended Role of Funder statement in your cover letter; we will change the online submission form on your behalf.*

**Answer:** We changed this as requested.

**Editor:**

4*. Thank you for stating the following in the Competing Interests section:*

*"I have read the journal's policy and the authors of this manuscript have the following competing interests:*

*Dr. Christina Schwens and Dr. Stefanie Klenner-Gastreich, two of the co-authors, are employed by Antech Diagnostics Germany which provided the biochemical, hematological, and thyroid hormone analyses included in this study. Dr. Daniel Müller is employed by SYNLAB MVZ Labor München Zentrum GbR and was responsible for the reverse T3 measurements. Ralf Mueller acted as a consultant or received support for studies or lectures from Artuvet, Bayer Animal Health, Ceva Animal Health, Ecuphar, Elanco Animal Health, Greer Laboratories, Heska Laboratories, Hill's, Royal Canin, MSD Animal Health, Nextmune, Antech, Virbac Animal Health and Zoetis. The authors declare that these affiliations had no influence on the design, execution, interpretation, or reporting of the study. No other conflicts of interest are declared."*

*Please confirm that this does not alter your adherence to all PLOS ONE policies on sharing data and materials, by including the following statement: "This does not alter our adherence to  PLOS ONE policies on sharing data and materials.” (as detailed online in our guide for authors* [*http://journals.plos.org/plosone/s/competing-interests*](http://journals.plos.org/plosone/s/competing-interests)*).  If there are restrictions on sharing of data and/or materials, please state these. Please note that we cannot proceed with consideration of your article until this information has been declared.*

*Please include your updated Competing Interests statement in your cover letter; we will change the online submission form on your behalf.*

**Answer:** We changed the COI Statement as follows:

Dr. Christina Schwens and Dr. Stefanie Klenner-Gastreich, two of the co-authors, are employed by Antech Lab Germany, which provided the biochemical, hematological, and thyroid hormone analyses included in this study. Dr. Daniel Müller is employed by SYNLAB MVZ Labor München Zentrum GbR and was responsible for the reverse T3 measurements. Ralf Mueller acted as a consultant or received support for studies or lectures from Artuvet, Bayer Animal Health, Ceva Animal Health, Ecuphar, Elanco Animal Health, Greer Laboratories, Heska Laboratories, Hill's, Royal Canin, MSD Animal Health, Nextmune, Antech, Virbac Animal Health and Zoetis. The authors declare that these affiliations had no influence on the design, execution, interpretation, or reporting of the study. They also do not alter our adherence to  PLOS ONE policies on sharing data and materials. No other conflicts of interest are declared.

**Editor:**

*5. We note that your Data Availability Statement is currently as follows: "All relevant data are within the manuscript and its Supporting Information files."*

*Please confirm at this time whether or not your submission contains all raw data required to replicate the results of your study. Authors must share the “minimal data set” for their submission. PLOS defines the minimal data set to consist of the data required to replicate all study findings reported in the article, as well as related metadata and methods (*[*https://journals.plos.org/plosone/s/data-availability#loc-minimal-data-set-definition*](https://journals.plos.org/plosone/s/data-availability#loc-minimal-data-set-definition)*).*

*For example, authors should submit the following data:*

*- The values behind the means, standard deviations and other measures reported;*

*- The values used to build graphs;*

*- The points extracted from images for analysis.*

*Authors do not need to submit their entire data set if only a portion of the data was used in the reported study.*

*If your submission does not contain these data, please either upload them as Supporting Information files or deposit them to a stable, public repository and provide us with the relevant URLs, DOIs, or accession numbers. For a list of recommended repositories, please see* [*https://journals.plos.org/plosone/s/recommended-repositories*](https://track.editorialmanager.com/CL0/https:%2F%2Fjournals.plos.org%2Fplosone%2Fs%2Frecommended-repositories/1/010f019d1e310623-ad2f698f-517f-4e0a-bba9-86dd07c06e8b-000000/v7dVRiukgk9Y0Fc8Dtc685d8-HvlVmGdmHcAo-x5c_E=255)*.*

*If there are ethical or legal restrictions on sharing a de-identified data set, please explain them in detail (e.g., data contain potentially sensitive information, data are owned by a third-party organization, etc.) and who has imposed them (e.g., an ethics committee). Please also provide contact information for a data access committee, ethics committee, or other institutional body to which data requests may be sent. If data are owned by a third party, please indicate how others may request data access.*

**Answer**: Thank you for this comment. We confirm that all data required to replicate the results of our study are provided in the Supporting Information files. The raw data underlying the analyses are included in S1–S3 Tables, corresponding to Age and Weight Correlation in healthy dogs, hypothyroid dogs, and dogs with NTIS, respectively.

These tables contain all values used for statistical analyses, including those underlying descriptive statistics and graphical representations.

All data are fully anonymized and comply with the journal’s data availability requirements. No ethical or legal restrictions apply to the sharing of these data.

**Editor:**

6*. If the reviewer comments include a recommendation to cite specific previously published works, please review and evaluate these publications to determine whether they are relevant and should be cited. There is no requirement to cite these works unless the editor has indicated otherwise.*

**Answer**: Thank you for this comment. We carefully reviewed the references suggested by the reviewer and assessed their relevance for our manuscript. Relevant publications were incorporated where appropriate.

**Response to the comments by Reviewer #1:**

***R1:***

*This is a clinically relevant study addressing the role of serum reverse triiodothyronine in dogs with hypothyroidism, nonthyroidal illness syndrome, and healthy controls. Overall, the study appears to be well designed and clearly presented; however, some points require clarification.*

**Answer:**

Thanks for your encouraging words. We are glad to hear that you find our study interesting and to present relevant data. In the following, we explain in greater detail how we incorporated each single comment in the manuscript.

***R1:***

*Line 16: Authors write that “tT4 is low in 30.2 – 52.5% of dogs with nonthyroidal illness syndrome (NTIS)”. This is not in agreement with the ALIVE criteria, “the syndrome is typically characterized by decreased circulating total T4 concentrations.”, therefore in all dogs and non only in some of them (Niessen et al. Agreeing Language in Veterinary Endocrinology (ALIVE): Hypothyroidism, Hyperthyroidism, (Euglycaemic) Diabetic Ketosis/Ketoacidosis, and Diabetic Remission-A Modified Delphi-Method- Based System to Create Consensus Definitions. Vet Sci. 2026 Jan 1;13(1):35.*

**Answer**: Thank you for this important comment. We have revised the respective paragraph in accordance with the ALIVE criteria. In particular, we clarified the distinction between non-thyroidal illness (NTI) and non-thyroidal illness syndrome (NTIS) and adapted the wording to reflect that decreased tT4 concentrations are a defining feature of NTIS, while the reported percentages refer to dogs with NTI.

“Diagnosing hypothyroidism by tT4 analysis is challenging as tT4 is not only low in hypothyroidism, but also in 30.2 in up to 52.5% of dogs with nonthyroidal illness (NTI) (3-5).”

***R1:*** *Line 18: I suggest so replace “ can differentiate” with “can be helpful in differentiating”*

***R1:*** *Line 20: replace “an” with “a”*

***R1:*** *Line 22: “All dogs…was conducted”. This sentence is not necessary and can be removed*

***R1:*** *Lines 30-31 ”The ROC area under the curve for rT3 was 0.981 (95% CI = 0.951-1.010).”The sentence is incomplete; it should explain what the ROC curve was used for.*

***R1:*** *Line 36 and 37: “using HPLC-MS/MS”. This is not a conclusion, this sentence should be in the M&M*

**Answer:** We have thoroughly revised and completely updated the abstract to better reflect our findings and comply with the journal's guidelines.

***R1:*** *Line 43. I suggest to replace “diseases” with “conditions”*

**Answer:** We replaced the term accordingly

***R1:*** *Lines 45-47 “Diagnosing hypothyroidism by tT4 analysis is challenging as tT4 is not only low in hypothyroidism, but also in 31% of dogs with nonthyroidal illness syndrome (NTIS).”See my comment above. Do not confuse non-thyroid disease with nonthyroidal illness syndrome. In the definition of nonthyroidal illness syndrome, the T4 is usually low*

**Answer**: Thank you for this important clarification. We have revised the respective sentence and now clearly distinguish between non-thyroidal illness (NTI) and non-thyroidal illness syndrome (NTIS) in accordance with the ALIVE criteria. Specifically, we used the definition of NTI when referring to the reported percentages of dogs with decreased tT4 concentrations and adjusted the wording accordingly to avoid confusion with NTIS, in which decreased tT4 is a defining feature.

“Diagnosing hypothyroidism by tT4 analysis is challenging as tT4 is not only low in hypothyroidism, but also in 30.2 in up to 52.5% of dogs with nonthyroidal illness (NTI).”

***R1:*** *Line 123: “assessment of tT4” Indicate the analytical performance of the method for measuring tT4 and TSH, also indicating the limit of quantification of the method. Tables show tT4 values as low as 0.13 ng/dl. Is the method linear at those tT4 concentrations?*

**Answer**: The lower limit of quantification for T4 measurement in the laboratory is 0.12 µg/dl and was determined in accordance with the ASVCP quality assurance guidelines. The analytical performance of tT4 and TSH was added in the paper.

***R1:*** *Lines 220-226: “Median rT3 concentration….in dogs with hypothyroidism and healthy controls” please report the data relating to fT4 in the same descriptive manner.*

**Answer**: Thank you for this comment. Free T4 (fT4) was measured in dogs with hypothyroidism and dogs with non-thyroidal illness (NTI), but not in healthy control dogs. Due to cost considerations and the limited clinical relevance of fT4 measurement in healthy animals, this parameter was not determined in this group. Therefore, corresponding descriptive data for fT4 in healthy controls are not available.

***R1:*** *Discussion: The discussion is good. However, there are some studies in which rT3 has previously been evaluated in dogs. It would be appropriate to cite them. E.g. Wood et al. Influence of isoflurane general anesthesia or anesthesia and surgery on thyroid function tests in dogs. J Vet Intern Med. 2009 Jan-Feb;23(1):7-15. Giunti et al Retrospective evaluation of circulating thyroid hormones in critically ill dogs with systemic inflammatory response syndrome. J Vet Sci. 2017 Dec 31;18(4):471-477.*

**Answer**: Thank you for your comments. We have revised both the Introduction and the Discussion accordingly. In the Introduction, we now include previous studies reporting rT3 measurements in dogs, including Wood et al. (2009) and Giunti et al. (2017), to provide context. In the Discussion, we specifically reference the recent abstract by Corsini et al. (2025) regarding rT3 in dogs suspected of hypothyroidism. The text has been updated to reflect these studies while maintaining that comparative studies directly assessing rT3 in dogs with hypothyroidism versus NTIS are still lacking.

**Response to the comments by Reviewer #2:**

***General comments***

***R2:*** *I would like to thank the authors for sharing these interesting and original data and compliment them on their work. The manuscript presents relevant data on the use of rT3, including validation of a new assay in dogs, definition of a reference interval, and a diagnostic accuracy study for hypothyroidism. However, in my opinion, many issues remain to be clarified, and the manuscript should be thoroughly revised.*

**Answer:** Thanks for your encouraging words. We are glad to hear that you find our study interesting and to present relevant data. We have carefully considered your feedback and decided to fundamentally revise the manuscript, or rather, to revise large portions of it. In the following, we explain in greater detail how we incorporated each single comment in the manuscript. ***R2:*** *Materials and Methods and the Results sections must be revised (see specific comments) to better adhere to the STARD guidelines for reporting diagnostic accuracy studies (*[*https://www.equator-network.org/reporting-guidelines/stard/*](https://www.equator-network.org/reporting-guidelines/stard/)*).*

**Answer:** Thank you for this comment. We have revised the Materials and Methods and Results sections to better comply with the STARD 2015 guidelines.

***R2:*** *In the Abstract and Results sections, I strongly suggest adding effect estimates (e.g., median differences) with 95% confidence intervals for both statistically significant and non-significant results. This approach gives more precise information to the reader, allowing for a better interpretation of the results beyond the P value.*

**Answer**: We have included the necessary statistical values throughout the paper. For normally distributed data (weight), we have reported the means with 95% confidence intervals, and for non-normally distributed data, we have reported the medians with the first and third interquartile ranges. The detailed statistical calculations are explained in the methods section.

***R2:*** *I suggest re-assessing the Discussion to better focus on the many results reported in the study, rather than reporting a general discussion of the pathophysiological mechanisms, which seems beyond the scope of the manuscript. Furthermore, some results require a more detailed discussion.*

**Answer**: Thank you very much for this excellent comment. We have revised the structure of the paper in line with your suggestions (Part 1: Establishment of the method and reference range; Part 2: Diagnostic performance of rT3 in distinguishing between NTIS and hypothyroidism, including a comparison of rT3 and fT4d) and have restructured the discussion accordingly to better focus on the important points. We have covered some of the general pathophysiological principles in the introduction and, in the discussion, have sought to focus on the key pathophysiological points for distinguishing between NTIS and hypothyroidism, as well as the diagnostic differences between fT4d and rT3. We have also expanded the discussion to discuss some points in detail (such as the comparison between rT3 and fT4d), as you suggested.

***R2:*** *I also suggest reviewing the most recent literature, as many of the citations are outdated. Indeed, reported citations are appropriate, but integration with the most recent publications is desirable.*

**Answer:** Thank you for this comment. We have reviewed the most recent literature and updated the manuscript accordingly and integrated recent publications alongside the previously cited studies to ensure the manuscript reflects current knowledge in the field.

***R2:*** *Regarding the literature search, I suggest defining how it was conducted. Rather than simply stating ‘there are no studies’ or ‘based on the author's knowledge’, it is preferable to add a line that defines how the search was performed (https://onlinelibrary.wiley.com/doi/10.1111/vru.12748).*

**Answer:** We added that information**.**

***R2:*** *For example, instead of lines 73-74 ‘Comparative studies on rT3 measurement in dogs are not currently available’, the authors can state ‘The following databases (XX, XX and XX) have been searched with the following keywords XX XX on [date, eg 01/01/2020], and no other reports of XX have been found’ or a similar formulation. This also allows the authors to modify the discussion. Whenever referencing in the Discussion (or any other section) to this, you can use statements like "Based on the literature search, this is the first time ..." or similar (see lines 254 and 273).*

**Answer**: Thank you for your comments. We have revised both the Introduction and the Discussion accordingly. In the Introduction, we now include previous studies reporting rT3 measurements in dogs, including Wood et al. (2009) and Giunti et al. (2017), to provide context. In the Discussion, we specifically reference the recent abstract by Corsini et al. (2025) regarding rT3 in dogs suspected of hypothyroidism. The text has been updated to reflect these studies while maintaining that comparative studies directly assessing rT3 in dogs with hypothyroidism versus NTIS are still lacking.

***Specific comments***

**Abstract**

***R2:*** *Line 29: Median value is 2.5, while the lower limit of the range is <5. I am not sure if this is the correct way to report these results. If you define values <5 as equal to 2.5 for statistical reasons (line 162-163), you should probably report the lower limit of the range equal to 2.5. Please check and, if the same issue occurs, report it in the results section.*

**Answer**: Thank you for this comment. As you suggested, we have decided to set all values <5 to 2.5 so that we can carry out statistical calculations.

“RT3 values below the detection limit of 5 were reported as 2.5 as done in other comparable studies (43).”

***R2:*** *Line 32-33: Please add the confidence interval for the sensitivity and specificity*.

**Answer:** Done

**Introduction**

***R2:*** *Line 46: When citing multiple studies, you should report the results of all these studies. There are different possible approaches. First, you can cite all the results (e.g., 31%, xx %, and xx %); second, you can just report the range (e.g., 31% to xx%, or xx% to 31%), as you did in line 65; finally, if you choose this percentage because it is the highest one reported, clearly state this (e.g., in up to 31%). Of note, you reported a range of prevalence in the abstract (line 16).*

**Answer:** Thank you for this comment. We have revised the text to clarify the reported prevalence, now indicating that the cited value represents the highest reported percentage among the referenced studies.

***R2:*** *Line 47: I suggest revising this statement. Even though a clear definition of NTIS is difficult to derive solely from T4 measurements (which is the definition of NTIS?), it is generally accepted that low T4 is a criterion for defining the occurrence of NTIS in veterinary medicine, since we do not routinely measure T3. If not, all dogs with any disease should be considered as affected by NTIS. In this context, it is more appropriate to use the definition ‘non-thyroidal illness’ (without syndrome) or simply ‘concurrent diseases’, with the second one probably avoiding some confusion between ‘non-thyroidal illness’ and ‘non-thyroidal illness syndrome’.*

**Answer:** Thank you for this important comment. We have revised the respective paragraph and the whole paper in accordance with the ALIVE criteria. In our study, we included dogs with decreased tT4 concentrations, which we classified as NTIS. We have used both terms, non-thyroidal illness (NTI) and non-thyroidal illness syndrome (NTIS) inconsistently in the first version of the paper but have now emphasized NTIS throughout the manuscript to reflect the inclusion criteria and avoid confusion.

***R2:*** *Line 48: ‘Stimulation’ instead of ‘response’. Also, I guess the definition of "non-invasive" could be called into question. Indeed, thyroid scintigraphy is certainly demanding, but not necessarily invasive (see comments on line 53). I suggest using ‘gold standard among the blood tests’, or something similar.*

**Answer:** We have revised the wording, replacing “response” with “stimulation” and removed the term “non-invasive.” Additionally, we now describe the TSH test as the “gold standard among the blood tests” to better reflect its role.

***R2:*** *Line 48-51: Please check the most recent literature:* [*https://onlinelibrary.wiley.com/doi/10.1111/jsap.13290*](https://onlinelibrary.wiley.com/doi/10.1111/jsap.13290)

**Answer:** We have cited the relevant literature and revised the paragraph accordingly.

***R2:*** *Line 53: Is it always the case? It probably depends on the protocol used, but I guess that thyroid scintigraphy can also be performed under sedation.*

**Answer:** We have revised the text to clarify that thyroid scintigraphy can be performed under different protocols, including sedation. Relevant references have been added to support this statement.

“Scintigraphy is a further possibility to distinguish hypothyroid from healthy dogs (11, 12). Due to limited availability of this methodology and a mandatory general anaesthesia (although some protocols allow sedation (13, 14)), this is not a suitable method for general veterinary practice (9).”

***R2:*** *Line 61: see comment on line 46 about the use of percentage extrapolated from the literature.*

**Answer:** We have revised the text to clarify the reported prevalence, now indicating that the cited value represents the highest reported percentage among the referenced studies.

***R2:*** *Line 58-63: Free T4-ED is not even mentioned among possible diagnostic tests. Since it is usually considered the most accurate test among the non-dynamic tests, I guess it is worth mentioning. It is even more important, given that the authors compared rT3 and fT4-ED in their study.*

**Answer:** Thank you for this important suggestion. We have added a section on free T4 measured with (fT4d) or without (fT4a) equilibrium dialysis in the Introduction, highlighting its role as the most accurate non-dynamic test for hypothyroidism and providing a detailed explanation.

“Free T4 (fT4), which represents approximately 1 % of total T4, is considered to be the biologically active form of thyroxine, that better reflects the true thyroid status (16). FT4 can be measured by analogue methods (immunoassays or RIA; fT4a) or with equilibrium dialysis (fT4d) (3, 4). In general, measurement of fT4 shows better specificity of 97% (fT4a) and 90 up to 100% (different methods fT4d) than tT4 for diagnosing hypothyroidism (17). However diagnostic sensitivity is not that high (80% fT4a and 71 up to 96% fT4d), measurement of fT4d is considered the single best diagnostic test for canine hypothyroidism as fT4d concentrations are less commonly influenced by drugs (17, 18). Furthermore, the measurement of fT4d is also unaffected by T4 autoantibodies (19). In contrast to older studys that reported fT4 to be less influenced by NTI (3) the study of Bennain showed values of fT4 under the reference values in 89.1% (fT4a) and 57.1% (fT4d) in dogs with NTIS (19). Furthermore, high cost and limited availability of equilibrium dialysis, making fT4 measurement a second-line diagnostic test for hypothyroidism (9, 18).”

***R2:*** *Line 66: Since rT3 is the core of the study, a brief description of rT3 is due before describing its use in humans and veterinary medicine. Furthermore, is it routinely used in humans?*

*I would consider scaling back the assumption about the usefulness of rT3 in humans: ‘has been suggested as a useful test to…’ instead of ‘is useful’?*

**Answer:** We have revised the Introduction to include a brief description of rT3 before discussing its use in humans and veterinary medicine. The text now also reflects a more cautious statement regarding its clinical utility, using “has been suggested as a useful test” instead of a definitive claim.

“Reverse triiodothyronine (rT3) is an inactive metabolite of thyroxine (T4) produced by inner ring deiodination via deiodinase enzymes. In men its formation increases during illness or stress as part of adaptive changes in thyroid hormone metabolism, but routine clinical measurement of rT3 is not established as a standard diagnostic test in humans (23, 24). In humans, decreased rT3 concentrations have been reported in hypothyroidism, whereas increased rT3 concentrations are observed in NTIS. Measurement of rT3 has therefore been proposed as an additional parameter to differentiate hypothyroidism from non-thyroidal illness syndrome (NTIS) in men (25-30).”

**R2*:*** *Line 69-73: Please specify if total T3 (tT3) or free T3 (fT3).*

**Answer:** We have clarified throughout the manuscript that all references to T3 refer to total T3 (tT3).

***R2:*** *Line 70: ‘Lower’ instead of ‘low’*

**Answer:** Thank you for picking that up, we changed it accordingly.

***R2:*** *Line 75: I suggest adding the validation of the assay as an aim of the study. It is an important result reported by the authors, but not adequately highlighted. Also, it seems that another aim of this study could be to compare rT3 and fT4-ED (see comment on lines 58-63).*

***R2:*** *Line 78: The study's aims should be followed by the author's hypothesis, at least for the comparative part of the study (e.g., We hypothesized that rT3 accurately…).*

**Answer:** We changed to: Therefore, this study aimed first to establish and validate an HPLC-MS/MS assay for measurement of rT3 in healthy dogs with determination of a reference interval, and second to evaluate whether rT3 is a useful parameter to differentiate between dogs with hypothyroidism and NTIS. A further aim was to compare rT3 with fT4 measured by equilibrium dialysis (fT4d). We hypothesized that rT3 would be able to differentiate hypothyroid from NTIS dogs and show comparable or complementary diagnostic performance to fT4d.

**M&Ms:**

***R2:*** *The study design is not clearly stated. ‘Prospective’ is not a study design, simply describing how dogs were included time-wise. In my opinion, the study has two distinct parts that should be described appropriately without overlap; the authors did so in the results section but not in the MMs section. The first part involves assay validation and the definition of a reference interval; the second part of the study is a diagnostic accuracy study assessing the performance of rT3. I recommend consulting STARD guidelines for reporting diagnostic accuracy studies,* [*https://www.equator-network.org/reporting-guidelines/stard/*](https://www.equator-network.org/reporting-guidelines/stard/)*. The M&M section should be revised accordingly.*

**Answer**: We thank the reviewer for this important comment and for referring us to the STARD guidelines. The Materials and Methods section (and all following sections) has been revised accordingly to better distinguish the two components of the study: (1) assay validation and establishment of a reference interval, and (2) evaluation of the diagnostic performance of rT3 in dogs with hypothyroidism and NTIS. In addition, the wording describing the study as “prospective” has been revised to avoid implying this alone represents the study design. The revised section now more clearly describes the study structure, participating institutions, ethical approval, and recruitment period.

***R2:*** *The main problem here is the definition of the reference standard: there is no specific test against which to compare rT3, but the proposed composite reference standard is somewhat acceptable from a clinical perspective. Still, this limitation should be clearly stated in the discussion, as you partially did in lines 336-340.*

**Answer:** We thank the reviewer for this important comment. We agree that no single definitive reference standard for canine hypothyroidism or NTIS exists and that the diagnosis in clinical practice is typically based on a combination of clinical findings, routine thyroid testing, and follow-up evaluation. We therefore used a composite reference standard to classify dogs in the respective study groups. This limitation has now been stated more explicitly in the Discussion section, including the potential impact on the interpretation of the diagnostic performance of rT3.

***R2:*** *Line 86-90: The original population from which dogs were included should be better defined, as well as the process leading to the inclusion of dogs in the groups. For example, what were the criteria used to consider a dog a potentially eligible participant (e.g., all dogs presented in the selected period in which a thyroid panel was performed)? Have you decided a priori how many dogs to include in each group, or do you include all dogs that meet the appropriate criteria?*

*Related to this, it seems that healthy dogs were included differently from the dogs used in the diagnostic accuracy study.*

**Answer:** In the revised version, we have defined the inclusion criteria for the groups in detail and included all the points you listed. The number of patients was based on a power calculation, which indicated a minimum sample size of 15–20 dogs per group.

***R2:*** *Line 92: Pruritus is not a clinical sign of hypothyroidism. Indeed, it can be present if there is concurrent pyoderma, but it should not be included among clinical signs suggesting hypothyroidism.*

**Answer:** You are of course right. We have removed pruritus from the list of clinical signs suggesting hypothyroidism, as it is not a primary sign of the disease.

***R2:*** *Line 97-98: What does ‘moderate to severe’ underlying disease mean? Which criteria were used to define a disease as moderate to severe?*

*Furthermore, how was hypothyroidism ruled out in NTIS dogs showing low tT4 and high TSH?*

**Answer:**. Thank you for those important points. We have adopted the classification criteria ‘mild to severe’ from Kantrowitz’s study and have now formulated them in the text accordingly.

We have explained in more detail in the text how to rule out hypothyroidism:

“...Hypothyroidism was ruled out in all dogs based on a combination of history, clinical assessment and laboratory findings. In particular, dogs did not show clinical signs consistent with hypothyroidism, and the underlying disease was considered sufficient to explain the altered thyroid parameters... Only dogs with a decreased tT4 and elevated TSH were included, while others were excluded.”

***R2:*** *Line 106: Whenever drug administration is used as an exclusion criterion, the time should be clearly stated. Was only ongoing treatment considered, or also previous treatment? In the second case, for how long had the drug to be discontinued before inclusion?*

**Answer:** Thank you for this important comment. Dogs receiving ongoing treatment known to affect thyroid hormone concentrations were excluded. However, no predefined discontinuation period for previous medication was applied, as the study population included both dogs with acute conditions (e.g., pericardial effusion, xylitol intoxication) and dogs with chronic diseases receiving prior treatment. This has now been clarified in the manuscript.

„Only ongoing treatments were considered for exclusion; no predefined discontinuation period for prior medication was applied, as the study population included both dogs with acute conditions and dogs with chronic diseases receiving previous treatment.”

***R2:*** *Line 108: Were TKIs included in the list of drugs leading to exclusion from the study? If not, can you please explain why?*

**Answer:** Only two dogs with neoplasia (transitional cell carcinoma of the bladder and a high-grade mast cell tumor) were included in the study population, and neither of these dogs had received tyrosine kinase inhibitors. The use of tyrosine kinase inhibitors was not defined as an exclusion criterion a priori.

***R2:*** *Line 121: The commercial name of the profile is irrelevant; I would simply report the parameter measured in each dog (e.g, ‘complete blood count, serum chemistry profile including……, and total T4 and TSH were measured by Antech Lab’)*

**Answer:** Done. “In all participants (healthy dogs, dogs with hypothyroidism or NTIS), a complete blood count, serum biochemistry profile, total T4 (tT4), and TSH were measured by Antech Lab Germany.”

***R2:*** *Line 130: Please specify the assays used to measure tT4 and TSH. I guess the lab used Siemens Immulite canine T4 and canine TSH, but it should be clearly stated. Also, specify if the reported intervals were calculated by the Lab.*

**Answer**: You are, of course, correct. The tT4 and TSH levels were measured using the Immulite system. We have provided details of the exact method and assay performance as well as the reported reference intervals.

***R2:*** *Line 133-141: I would consider reporting this section as the results of the validation of the assay, rather than MM. These data are relevant, given that this assay was not previously validated in dogs (see line 75).*

**Answer**: Thank you for the good comment. We have included the validation of the rT3 assay in the results section and made the necessary adjustments.

***R2*** *Line 143-151: It is not clear to me why all these parameters, except for FT-ED, were measured in both groups. Indeed, the results about tT3, T4AA, T3AA, and TgAA were not analyzed (not even descriptively) nor discussed. As it is, they seem quite out of context. Nonetheless, some of this data could probably be analyzed in an interesting way. For example, based on what is described in lines 66-69, has the author considered the possibility of analyzing the T3 to rT3 ratio in these dogs? It clearly was not planned a priori, but the authors could conduct it as a post hoc analysis and appropriately report it in the Statistical Analysis section.*

**Answer**: We thank the reviewer for this valuable comment. The additional thyroid-related parameters were included to further characterize thyroid status and support the clinical differentiation between hypothyroidism and NTIS as part of the extended thyroid panel performed by the Veterinary Diagnostic Laboratory at Michigan State University. We agree that these parameters were not analyzed in detail within the scope of the present study, and we have clarified this intention in the Materials and Methods section to improve context and transparency.

We also appreciate the reviewer’s suggestion regarding analysis of the T3/rT3 ratio. Although this may provide additional insight into thyroid hormone metabolism in dogs with hypothyroidism and NTIS, this analysis was not predefined in the study design and was therefore not included in the present manuscript. In addition, approximately one-third of the dogs with NTIS and hypothyroidism showed T3 concentration reported as zero from Michigan State University, resulting in a T3/rT3 ratio of 0, while the remaining subgroup sizes were considered too small for meaningful interpretation. We therefore believe that evaluation of the T3/rT3 ratio would require a dedicated follow-up study with a larger study population and an appropriate statistical design.

***R2:*** *Line 154: Why were the data expressed as median and range? Was it due to non-normal distribution? How was the distribution of the data assessed? Furthermore, please be aware that range is not a measure of dispersion for non-normally distributed data; these should be described using other measures (e.g., the interquartile range). For this reason, IQR provides a better assessment of the variability of a dataset compared to range.*

**Answer**: Thank you very much for your comment. We have revised the statistics. The data were tested for normality using the Anderson-Darlin test. Normally distributed data (which were only present for the dogs’ weights) were reported using the mean and the 95% CI, whilst the non-normally distributed data were presented using the median and the IQR. We have chosen the following wording for the Materials and Methods section:

“The Anderson-Darling test was used to assess the normality of the distribution of age, weight and all analysed laboratory data (tT4, TSH and rT3) within the 3 groups. Normally distributed data (weight) were tested for significance using ANOVA and the Tukey test for multiple comparisons, whilst non-normally distributed data (age, tT4, TSH, rT3) were tested using the Kruskal-Wallis test followed by the Steel-Dwass-Critchlow-Fligner test for multiple comparisons. Normally distributed data were reported as means with 95% confidence intervals (weight), whilst all other non-normally distributed data are reported as medians with interquartile ranges [first quartile: Q1, third quartile: Q3].“

***R2:*** *Line 156-157: I suggest to clearly state which method was used here, and not in the results (line 194-195). E.g., ‘…was calculated using a robust untransformed…based on the…’*

**Answer**: Done

***R2:*** *Line 159: This sentence is not clear. The authors must describe here in the MM the analysis performed. E.g., ‘In healthy dogs, rT3 values were compared between young (healthy dogs <6 years) and old dogs (healthy dogs >/=7 years) using…’. Also, if the authors want to explore the correlation between age and rT3 values, it could be interesting to perform a correlation analysis (e.g. Spearman’s correlation).*

**Answer:** We have incorporated your suggested changes and have also carried out a Spearman's correlation analysis between age and rT3 values, which we have described accordingly.

***R2:*** *Line 161: The ROC curve analysis was performed for both rT3 and FT4-ED, but the latter is not reported here. Please add it.*

**Answer:** We added the data and the graph for fT4d comparison of hypothyroid dogs and dogs with NTIS.

***R2:*** *Line 164: See also the general comment of estimates of effects and confidence interval. Please add this part in the statistical section.*

**Answer:** We have added the required information to the statistical section and worded it as follows:

“Normally distributed data were reported as means with 95% confidence intervals (weight), whilst all other non-normally distributed data are reported as medians with interquartile ranges [first quartile: Q1, third quartile: Q3].“

***Results:***

***R2:*** *Line 167-168: I strongly suggest adding a flowchart at the beginning of this section, to highlight how the population in the diagnostic accuracy part of the study was created, as suggested by the STARD guidelines* [*https://www.equator-network.org/wp-content/uploads/2015/03/STARD-2015-flow-diagram.pdf*](https://www.equator-network.org/wp-content/uploads/2015/03/STARD-2015-flow-diagram.pdf)*. Also, considering dogs were included from many centers, the number of dogs included in each center (Haar, University and lab-referred) should be clarified.*

**Answer**: Thank you for this helpful suggestion. We have created a STARD-compliant flowchart illustrating the inclusion and exclusion process of dogs in the diagnostic accuracy part of the study. The flowchart has been added as Figure 1, is referenced in the manuscript, and has also been uploaded as a separate figure file in accordance with journal requirements.

In addition, we have clarified the number of dogs included from each center (AniCura Veterinary Clinic Haar, Small Animal Clinic of the University of Munich, and the commercial laboratory) within the flowchart to improve transparency.

***R2:*** *Line 176-181: Please avoid the use of ‘tends to’ or reporting population as younger or older if statistical significance is not achieved. To make these comparisons clearer, I suggest summarizing data about sex, age and body weight in a Table, describing only the comparisons with median difference and confidence intervals in the text. E.g., ‘No age difference was observed between hypo and healthy dogs (MD = xxx, 95% CI xx-xx, p= xxx) nor between NTIS and hypo dogs (MD = xxx, 95% CI xx-xx, p= xxx), while NTIS were older compared to healthy dogs (MD = xxx, 95% CI xx-xx, p= xxx),’. This way, the reader has all data to critically analyze the results reported, without the use of speculative terms such as ‘tends to’.*

*The same suggestions apply to all the Results section.*

**Answer**: Thank you for this helpful suggestion. We have revised the manuscript accordingly by removing non-significant descriptive terms such as “tended to” and now report only statistically supported comparisons.

Age differences are now presented using median differences with corresponding IQR and p-values. In addition, we have created a new tables (Table 2 & 3) summarizing age and body weight across all groups to improve clarity and transparency of the data presentation.

***R2:*** *Figure 1: See previous comment. I suggest to graphically underline the significant differences between NTIS and healthy dogs.*

**Answer:** Thank you for the suggestion. The significant differences between NTIS and healthy dogs are now graphically highlighted in Figure 2 by inclusion of the corresponding p-values.

***R2:*** *Line 188: I would put the results of the validation here, along with the ones regarding the reference interval, in a dedicated paragraph. E.g., ‘Validation of rT3 assay and reference interval in healthy dogs.’*

**Answer**: Thank you for this helpful suggestion. We have revised the manuscript accordingly by creating a dedicated paragraph entitled “Validation of rT3 assay and reference interval in healthy dogs,” in which the results of the assay validation and the reference interval determination are now presented together to improve clarity and structure.

***R2:*** *Table 1, 2, or 3 and lines 200-209: I suggest moving these three tables in the Supplementals. Even if the possibility to look at single dog is interesting, I do not believe it is really required in the main texts. Rather, I would summarize the descriptive analysis (median, IQR, range) regarding T4, TSH, rT3, and FT4 in the three groups in a single table, reporting the results of the comparisons in the text (see previous comments).*

*Furthermore, it could be interesting to know how many dogs (percentage) in hypo and NTIS group showed rT3 and fT4-ED values below (which means a positive test), within, or above (which means a negative test) the reference interval. In a diagnostic accuracy study, this is more valuable than a comparison between median values of healthy dogs and hypo/NTIS dogs: to extremize the concept, median values of dogs in hypo/NTIS groups could hypothetically be lower compared to healthy ones, but still, most of the single value of each dog could remain within the reference interval.*

**Answer**: Thank you for this valuable suggestion. We agree that a clearer focus on group-level summaries and diagnostic interpretation improves the readability and clinical relevance of the manuscript.

We have therefore revised the manuscript accordingly. Tables 1–3 have been moved to the Supporting Information and are now provided as S1–S3 Tables (Age and Weight Correlation in healthy dogs, hypothyroid dogs, and dogs with NTIS, respectively). These tables are available in the Supporting Information section and contain detailed individual-level data, including underlying diseases and severity of NTIS, which are relevant for interpretation but not essential for the main manuscript.

In the revised manuscript, Table 1 presents baseline and laboratory characteristics of healthy dogs, dogs with hypothyroidism, and dogs with NTIS. Table 2 summarizes baseline characteristics of the three groups. Table 3 presents demographic characteristics (age and body weight) of healthy, hypothyroid, and NTIS dogs, including p-values. Table 4 provides pairwise statistical comparisons between the three groups (p-values). Table 5 reports the classification of hypothyroid and NTIS dogs based on rT3 and fT4d reference intervals, showing the proportion of dogs below, within, and above the respective reference ranges.

This revision improves the structure of the manuscript by emphasizing group-level summaries while incorporating clinically relevant diagnostic interpretation at the individual level, as suggested.

***R2:*** *Figure 2: I suggest to graphically show the reference interval, to emphasize the distribution of the rT3 below, within or above the RI in the three groups.*

**Answer**: Thank you for this helpful suggestion. We have revised Figure 2 (now Figure 4) to include the reference interval for rT3, allowing visualization of values below, within, and above the reference range across the three groups. This improves the interpretability of the data at the individual level.

***R2:*** *Line 227: This sentence should be in the statistical analysis section.*

**Answer:** We have removed the sentence from the results section as requested.

***R2:*** *Line 228-232 and 238-242: Please add the confidence interval for sensitivity and specificity values.*

**Answer:** We added the CI interval as requested in the manuscript**.**

***R2:*** *Figure 3 and 4: I would consider joining these two figures, to better emphasize the comparison between the ROC curves of the different tests.*

**Answer:** Thank you very much for this valuable suggestion, which we have implemented as requested, now Figure 5.

**Discussion:**

***R2:*** *Line 250-253: The incipit of the discussion usually summarizes the most relevant results of the study. In my opinion, I would add here the validation of the assay and the better performance of the rT3 compared with fT4-ED.*

**Answer**: We added the requested information.

***R2:*** *Line 254-255: The authors stated there are no comparative studies, but later they discuss the results of Corsini et al. 2025 (line 294-315). I agree with the authors that there are no studies published on peer-reviewed journals (provided their literature search confirm this assumption – see general comments), since Corsini et al. is a conference presentation and those results are unpublished to date. However, this discrepancy is not adequately discussed. I would probably consider at least underlining the nature of the Corsini at al. study in the manuscript.*

**Answer**: We changed the text accordingly.

***R2:*** *Line 256-274: This entire paragraph is out of focus and should be removed from the discussion. This is merely a pathophysiological discussion about rT3 and NTIS, which is not related to the results reported by the authors. Indeed, the discussion must focus on the results presented in the study. This section could be moved, in a shorter and summarized version, in the Introduction to give the reader some information on what rT3 is and why it could be useful.*

**Answer**: We have revised the manuscript accordingly by removing the detailed pathophysiological discussion on rT3 and NTIS from the Discussion section, as it was not directly related to the results of the present study.

A concise and focused description of rT3 and its potential diagnostic relevance has now been incorporated into the Introduction to provide appropriate background information for the reader.

***R2:*** *Line 276-277: see previous comments*

***R2:*** *Line 279-281: These are results, that should not be repeated in the discussion. Rather, discuss the implication of these results, if considered relevant.*

**Answer**: We changed the text accordingly.

***R2:*** *Line 281-283: That’s true, but is this clinically relevant? We do not use any of this test to discriminate healthy and hypothyroid dogs, but rather to discriminate dogs with clinical signs (or laboratory abnormalities) due to hypothyroidism or due to NTIS.*

**Answer:**  Thank you for this important comment. We agree that clinical relevance primarily lies in differentiating hypothyroid dogs from dogs with NTIS rather than from healthy animals. Therefore, we revised the corresponding section of the manuscript.

***R2:*** *Line 284-285: Please, go into details. For example, you should comment on the discrepancy in the percentage of dogs with NTIS with high rT3 (which must be added in the results, see previous comments) compared to humans. Which are the possible explanations? Maybe assessing tT3 and rT3 (see previous comments) can give you some idea.*

**Answer:** Thank you for this insightful comment. We have expanded the Discussion to address the discrepancy between the proportion of dogs with NTIS showing increased rT3 concentrations and findings reported in humans. We now discuss potential explanations, including species-specific differences in thyroid hormone metabolism, disease severity, and deiodinase activity.

***R2:*** *Line 290-293: This seems to me the most interesting and clinically relevant result of the present study. I would highlight it and discuss it properly.*

**Answer:** Thank you for this important comment. We agree that this represents one of the most clinically relevant findings of our study. We have revised the Discussion to highlight the superior diagnostic performance of rT3 compared to fT4d and expanded the discussion to better address its clinical implications, potential mechanisms, and limitations.

***R2:*** *Line 317: ‘is’ rather than ‘could be explained’*

**Answer:** You are right, we changed accordingly.

***R2:*** *Line 328-329: Please check the literature:* [*https://academic.oup.com/jvim/article/38/3/1345/8456062*](https://academic.oup.com/jvim/article/38/3/1345/8456062)

**Answer:** Done.

***R2:*** *Line 332: It may be true, but it is not explained why the sample size limits the interpretation of the Results. For example, smaller samples lead to decreased statistical power; of note, this study has no power analysis, and this is a limitation worthy to remember. The (hypothetical) low statistical power increase the risk of type II error (failing to detect a difference that exist); however, does not seem the case considering the authors were able to demonstrate their hypothesis. Another possible limitation related to the low statistical power, is that the confidence intervals could be very wide; however, since now confidence intervals are not reported for most of the results, I am not able to comment on this (and so is the hypothetical reader).*

**Answer**: Thank you for this valuable comment. We agree that the relatively small sample size represents a limitation of the present study and may reduce statistical power, thereby increasing the risk of type II error. Higher patient numbers would have been preferable to further strengthen the robustness of the results. Nevertheless, despite the limited sample size, statistically significant differences between groups were detected, supporting the validity of the observed findings. In addition, confidence intervals have now been added for the majority of relevant results to improve the interpretation and transparency of the statistical analyses.

***R2:*** *Line 336-339: See previous comments, reported here - The main problem here is the definition of the reference standard: there is no specific test against which to compare rT3, but the proposed composite reference standard is somewhat acceptable from a clinical perspective.*

**Answer:** We have revised the Discussion to more clearly address the limitations related to the reference standard. In particular, we now acknowledge the use of a composite reference standard and the absence of definitive gold standard testing, as well as the potential for misclassification.

We also revised the section on treatment response and follow-up measurements to provide a more cautious interpretation of these findings.

***R2:*** *Line 340-341: This is a result. The fact that rT3 was rechecked after treatment or after resolution of the concomitant diseases in a subset of dogs should be clearly described in the MM section/Study design, and these lines moved from the Discussion to the Results. Then, you can refer to the result to support you argumentation here.*

**Answer**: Thank you for this helpful comment. We agree that these findings represented results rather than discussion points. Therefore, the corresponding statements were removed from the Discussion section and are no longer discussed in the revised manuscript.

***R2:*** *Line 357-358: This is well discussed, but it could be even better to have a correlation analysis (see previous comments).*

**Answe**r: We carried out a Spearman rank correlation analysis and discuss the results accordingly.

***R2:*** *Line 361-364: Same comment as lines 250-253*

***R2:*** *Line 364-368: I would remove these lines. Generically stating the need of further studies is of no value. Rather, the authors could focus on the indication of performing more studies in* *dogs with different conditions or subclinical hypothyroidism, if they believe those are the settings that should be further investigated based on their results.*

**Answer:** Thank you for these helpful comments. We have revised the conclusion to better highlight the key findings of the study, particularly the superior diagnostic performance of rT3 compared to fT4d.

In addition, we have removed generic statements regarding the need for further studies and instead specified future research directions based on our results, including the evaluation of rT3 in dogs with different underlying conditions and in subclinical hypothyroidism, as well as the potential value of combined assessment of rT3 and tT3.

“Based on our findings, further investigations should focus on the evaluation of rT3 in dogs with varying disease severity, different underlying conditions associated with NTIS, and in cases of subclinical hypothyroidism, where diagnostic differentiation remains particularly challenging. In addition, combined assessment of rT3 and tT3 may provide further insight into thyroid hormone metabolism and should be explored in future studies.”

***References***

***R2:*** *Line 472: This citation should be revised (*[*https://doi.org/10.1093/jvimsj/aalaf018*](https://doi.org/10.1093/jvimsj/aalaf018)*). Please check the guidelines for citing conference abstracts.*

**Answer:** We revised the citation as requested.
