# Supplementary material for: Serum reverse triiodothyronine in dogs with hypothyroidism, nonthyroidal illness syndrome and healthy dogs
Source: PLoS One. 2026 Oct 5;21(10):e0357106. doi: 10.1371/journal.pone.0357106 (PMC13637879; doi:10.1371/journal.pone.0357106)
Supplement: Supplementary file 6 [file pone.0357106.s006.docx]

**Response to the Academic Editor Part 2**

**Journal Requirements:**

If the reviewer comments include a recommendation to cite specific previously published works, please review and evaluate these publications to determine whether they are relevant and should be cited. There is no requirement to cite these works unless the editor has indicated otherwise. 

Please review your reference list to ensure that it is complete and correct. If you have cited papers that have been retracted, please include the rationale for doing so in the manuscript text, or remove these references and replace them with relevant current references. Any changes to the reference list should be mentioned in the rebuttal letter that accompanies your revised manuscript. If you need to cite a retracted article, indicate the article’s retracted status in the References list and also include a citation and full reference for the retraction notice.

**Answer:**

We thank the Editor for these comments.

The recommended publication has been carefully reviewed and, as it was considered highly relevant to the present study, it has been incorporated into the Introduction, Discussion, and the reference list. Furthermore, the complete reference list has been carefully checked for accuracy and completeness. No retracted publications are cited in the revised manuscript. The reference list has been updated accordingly, and all changes are reflected in the revised manuscript.

**Reviewers' comments:**

Reviewer's Responses to Questions

**Comments to the Author**

1. If the authors have adequately addressed your comments raised in a previous round of review and you feel that this manuscript is now acceptable for publication, you may indicate that here to bypass the “Comments to the Author” section, enter your conflict of interest statement in the “Confidential to Editor” section, and submit your "Accept" recommendation.

**Andrea Corsini:** (No Response)

2. Is the manuscript technically sound, and do the data support the conclusions?

The manuscript must describe a technically sound piece of scientific research with data that supports the conclusions. Experiments must have been conducted rigorously, with appropriate controls, replication, and sample sizes. The conclusions must be drawn appropriately based on the data presented.

**Andrea Corsini:** Yes

3. Has the statistical analysis been performed appropriately and rigorously?

**Andrea Corsini:** Yes

4. Have the authors made all data underlying the findings in their manuscript fully available?

The [PLOS Data policy](http://track.editorialmanager.com/CL0/http:%2F%2Fwww.plosone.org%2Fstatic%2Fpolicies.action%23sharing/1/010f019f16e71860-49640be5-9269-482a-b0f9-ff4660b241d1-000000/NlzK0pXgWvxeTlgcrIJPBCM9qzpQWdf3eiA8NOs2LPA=258) requires authors to make all data underlying the findings described in their manuscript fully available without restriction, with rare exception (please refer to the Data Availability Statement in the manuscript PDF file). The data should be provided as part of the manuscript or its supporting information, or deposited to a public repository. For example, in addition to summary statistics, the data points behind means, medians and variance measures should be available. If there are restrictions on publicly sharing data—e.g. participant privacy or use of data from a third party—those must be specified.

**Andrea Corsini:** Yes

5. Is the manuscript presented in an intelligible fashion and written in standard English?
PLOS ONE does not copyedit accepted manuscripts, so the language in submitted articles must be clear, correct, and unambiguous. Any typographical or grammatical errors should be corrected at revision, so please note any specific errors here.

**Andrea Corsini:** Yes

6. Review Comments to the Author
Please use the space provided to explain your answers to the questions above. You may also include additional comments for the author, including concerns about dual publication, research ethics, or publication ethics. (Please upload your review as an attachment if it exceeds 20,000 characters)

**Andrea Corsini:** The authors adequately responded to many of my concerns. However, I have some further comments that I would like to see addressed before publication.

**Response to the comments by Andrea Corsini:**

I previously commented on the use of point estimates of effect (mean or median difference) with 95% CI as a tool to better describe their results, providing more information to the reader. However, I think the authors may have misunderstood my comment. Indeed, estimates of effect were not included (<https://www.acpjournals.org/doi/10.7326/0003-4819-114-6-515> or <https://www.bmj.com/content/292/6522/746>), although the authors stated that they had done so for “age”. Instead, the authors reported 95% CI together with the mean. The use of mean +/- 95% CI is possible, but not for describing the variability in the population from which the sample was drawn (see: <https://doi.org/10.1136/bmj.331.7521.903>), which is the usual purpose of descriptive statistics. This is also the rationale for using IQR when reporting medians. I suggest describing “weight” using mean ± standard deviation, which seems more useful than the standard error and derived variables, such as confidence intervals, in this specific setting.

**Answer:** We would like to sincerely thank the reviewer for the detailed explanations and apologize for the misunderstanding.

We have added the median and standard deviation to the paper to improve the descriptive presentation of weight.

In addition, in the group comparison, we have included the paired mean difference with a 95% CI for the normally distributed data (weight) and, for the non-normally distributed data (e.g., age), the paired differences calculated using the Hodges-Lehman location shift with a 95% CI. In addition to incorporating this data into the text, we have also added two additional tables (weight and age) with the exact numerical values to provide a clearer presentation.

Line 52: The AUC for fT4d is already reported in line 49. Please remove one of the two instances. I would report the exact value corresponding to the lower limit of the RI for fT4d.

**Answer:** done

Line 70: The term NTIS is used for the first time here, but it is not spelled out. This also occurs several times before line 105, in which the author spelled out NTIS. Also, NTI is still used to denote diseases not affecting the thyroid that could influence thyroid function; even if the distinction from NTIS is clearer and the use of NTI is appropriate, I believe that using a different term would help avoid any possible confusion between the syndrome leading to low T4 (NTIS) and concurrent non-thyroidal diseases (NTI).

**Answer:** We thank the reviewer for this helpful suggestion. The abbreviation NTIS has been spelled out at its first occurrence in the manuscript. To avoid potential confusion between nonthyroidal illness syndrome (NTIS) and concurrent non-thyroidal diseases, the abbreviation NTI has been removed and replaced by the term “non-thyroidal diseases” throughout the manuscript.

Line 105: I suggest using ‘humans’ instead of ‘men’, as the latter could be misleading and interpreted as referring specifically to the male sex.
**Answer:** Done.

Line 110: A paper validating rT3 measured with LC-MS/MS assay in healthy dogs has recently been published (<https://doi.org/10.1016/j.domaniend.2026.107024>). I believe it would be worth citing and discussing this study. For example, the reported reference interval (107–542 pg/mL) appears very similar to that described in the present study, despite LC-MS/MS being performed in two different laboratories. This seems promising, as it may suggest low inter-assay variability, particularly at the lower cut-offs.

**Answer:** We thank the reviewer for this valuable suggestion. The recently published study has been cited in both the Introduction and the Discussion. In the Discussion, we additionally compare the reported reference interval with our findings and discuss the comparable reference intervals obtained in two independent laboratories using LC-MS/MS, as suggested.

Line 197-198: It is commendable that the authors performed an a priori statistical power analysis to justify the target sample size. However, the power analysis itself should be described in sufficient detail in the statistical section. Specifically, the authors should report the assumed effect size, significance level (α), desired power (1 − β), statistical test used, and the software employed to perform the calculation.

**Answer:** We added the asked detail in the paper.

Lines 306-308: Please clarify the rationale for excluding the three outliers. Were they identified using a predefined statistical outlier detection method or excluded for analytical/clinical reasons? Furthermore, the statement that the selected reference interval "provided the most plausible results" is subjective and should be replaced with an objective description of the statistical method used to derive the final reference interval.
**Answer:** Thank you very much for this valuable comment. We identified the outliers using Tukey's test and have updated this information in the statistical section accordingly. We have also standardized the methodology for calculating reference intervals and removed the phrase “provided that the results are plausible,” as you pointed out.

Lines 321–345: I would consider reporting the sensitivity and specificity results for different cut-offs in a table. This would improve the interpretation and comparison of the different cut-offs.

**Answer:** Done.

Table 4: I think this table would be more informative and useful if the pairwise comparisons were reported as median differences with 95% confidence intervals instead of p-values.

**Answer:** We have added the requested data to the table. Because the data were not normally distributed group differences were summarized as explained in the statistics using the Hodge-Lehman location shift together with 95% confidence intervals.

Table 5: Are the authors sure that the term “negative test” is appropriate for results above the reference interval? Indeed, in clinical practice, a normal fT4ed or rT3 result is usually considered “negative”.

**Answer:** Thank you very much for your valuable comment. The terms “negative” and “positive” can be misleading in this context. We have therefore avoided using these terms and have presented the classification of the laboratory values in a purely descriptive manner.

7. PLOS authors have the option to publish the peer review history of their article ([what does this mean?](https://track.editorialmanager.com/CL0/https:%2F%2Fjournals.plos.org%2Fplosone%2Fs%2Feditorial-and-peer-review-process%23loc-peer-review-history/1/010f019f16e71860-49640be5-9269-482a-b0f9-ff4660b241d1-000000/z4U4j-IfNolpIpxMokoAldOnje3zt8c-yoyYpZbogW8=258)). If published, this will include your full peer review and any attached files.

If you choose “no”, your identity will remain anonymous but your review may still be made public.

**Do you want your identity to be public for this peer review?** For information about this choice, including consent withdrawal, please see our [Privacy Policy](https://track.editorialmanager.com/CL0/https:%2F%2Fwww.plos.org%2Fprivacy-policy/1/010f019f16e71860-49640be5-9269-482a-b0f9-ff4660b241d1-000000/Q6daFRIq5u6F1YQUwFQhIg0ot4g4LYyT5BcCIbjyVmM=258).

Reviewer #2: **Yes:** Andrea Corsini

[NOTE: If reviewer comments were submitted as an attachment file, they will be attached to this email and accessible via the submission site. Please log into your account, locate the manuscript record, and check for the action link "View Attachments". If this link does not appear, there are no attachment files.]

To ensure your figures meet our technical requirements, please review our figure guidelines: <https://journals.plos.org/plosone/s/figures>

You may also use PLOS’s free figure tool, NAAS, to help you prepare publication quality figures: <https://journals.plos.org/plosone/s/figures#loc-tools-for-figure-preparation>.

NAAS will assess whether your figures meet our technical requirements by comparing each figure against our figure specifications.
