# Supplementary material for: Serum reverse triiodothyronine in dogs with hypothyroidism, nonthyroidal illness syndrome and healthy dogs
Source: PLoS One. 2026 Oct 5;21(10):e0357106. doi: 10.1371/journal.pone.0357106 (PMC13637879; doi:10.1371/journal.pone.0357106)
Supplement: Peer review history

# Peer review: Decision Letter 0

**DOI:** 10.1371/journal.pone.0357106.r001

**Authors:** Benito Soto-Blanco

24 Mar 2026

Dear Dr. Ruß,

Thank you for submitting your manuscript to PLOS ONE. After careful consideration, we feel that it has merit but does not fully meet PLOS ONE’s publication criteria as it currently stands. Therefore, we invite you to submit a revised version of the manuscript that addresses the points raised during the review process.

Please submit your revised manuscript by May 08 2026 11:59PM. If you will need more time than this to complete your revisions, please reply to this message or contact the journal office at plosone@plos.org. When you're ready to submit your revision, log on to https://www.editorialmanager.com/pone/ and select the 'Submissions Needing Revision' folder to locate your manuscript file.

- A letter that responds to each point raised by the academic editor and reviewer(s). You should upload this letter as a separate file labeled 'Response to Reviewers'.
- A marked-up copy of your manuscript that highlights changes made to the original version. You should upload this as a separate file labeled 'Revised Manuscript with Track Changes'.
- An unmarked version of your revised paper without tracked changes. You should upload this as a separate file labeled 'Manuscript'.

If you would like to make changes to your financial disclosure, please include your updated statement in your cover letter. Guidelines for resubmitting your figure files are available below the reviewer comments at the end of this letter.

If applicable, we recommend that you deposit your laboratory protocols in protocols.io to enhance the reproducibility of your results. Protocols.io assigns your protocol its own identifier (DOI) so that it can be cited independently in the future. For instructions see: https://journals.plos.org/plosone/s/submission-guidelines#loc-laboratory-protocols. Additionally, PLOS ONE offers an option for publishing peer-reviewed Lab Protocol articles, which describe protocols hosted on protocols.io. Read more information on sharing protocols at https://plos.org/protocols?utm_medium=editorial-email&utm_source=authorletters&utm_campaign=protocols.

We look forward to receiving your revised manuscript.

Kind regards,

Benito Soto-Blanco, DVM, MSc, PhD

Academic Editor

PLOS One

Journal Requirements:

When submitting your revision, we need you to address these additional requirements.

1. Please ensure that your manuscript meets PLOS ONE's style requirements, including those for file naming. The PLOS ONE style templates can be found at

https://journals.plos.org/plosone/s/file?id=wjVg/PLOSOne_formatting_sample_main_body.pdf and

https://journals.plos.org/plosone/s/file?id=ba62/PLOSOne_formatting_sample_title_authors_affiliations.pdf

2. hank you for stating the following in the Acknowledgments Section of your manuscript:

"We thank the laboratory teams at Antech Lab GmbH and SYNLAB MVZ Munich GbR for their technical support. We also gratefully acknowledge the DVGD (Deutsche Veterinärmedizinische Gesellschaft für Dermatologie) for financial support through a research grant."

We note that you have provided additional information within the Acknowledgements Section that is not currently declared in your Funding Statement. Please note that funding information should not appear in the Acknowledgments section or other areas of your manuscript. We will only publish funding information present in the Funding Statement section of the online submission form.

Please remove any funding-related text from the manuscript and let us know how you would like to update your Funding Statement. Currently, your Funding Statement reads as follows:

"This study was financially supported (JR) by a research grant from the DGVD – Deutsche Gesellschaft für Veterinärdermatologie (German Veterinary Dermatology Society), which is gratefully acknowledged.

URL: https://www.dgvd.org/

Grant Number: 01/2022"

Please include your amended statements within your cover letter; we will change the online submission form on your behalf.

3. Thank you for stating the following financial disclosure:

"This study was financially supported (JR) by a research grant from the DGVD – Deutsche Gesellschaft für Veterinärdermatologie (German Veterinary Dermatology Society), which is gratefully acknowledged.

URL: https://www.dgvd.org/

Grant Number: 01/2022"

Please state what role the funders took in the study.  If the funders had no role, please state: "The funders had no role in study design, data collection and analysis, decision to publish, or preparation of the manuscript."

If this statement is not correct you must amend it as needed.

Please include this amended Role of Funder statement in your cover letter; we will change the online submission form on your behalf.

4. Thank you for stating the following in the Competing Interests section:

"I have read the journal's policy and the authors of this manuscript have the following competing interests:

Dr. Christina Schwens, one of the co-authors, is employed by Antech Germany GmbH, which provided the biochemical, hematological, and thyroid hormone analyses included in this study. Dr. Daniel Müller is employed by SYNLAB MVZ Labor München Zentrum GbR and was responsible for the reverse T3 measurements. Ralf Mueller acted as a consultant or received support for studies or lectures from Artuvet, Bayer Animal Health, Ceva Animal Health, Ecuphar, Elanco Animal Health, Greer Laboratories, Heska Laboratories, Hill's, Royal Canin, MSD Animal Health, Nextmune, Antech, Virbac Animal Health and Zoetis. The authors declare that these affiliations had no influence on the design, execution, interpretation, or reporting of the study. No other conflicts of interest are declared."

Please confirm that this does not alter your adherence to all PLOS ONE policies on sharing data and materials, by including the following statement: "This does not alter our adherence to  PLOS ONE policies on sharing data and materials.” (as detailed online in our guide for authors http://journals.plos.org/plosone/s/competing-interests).  If there are restrictions on sharing of data and/or materials, please state these. Please note that we cannot proceed with consideration of your article until this information has been declared.

Please include your updated Competing Interests statement in your cover letter; we will change the online submission form on your behalf.

5. We note that your Data Availability Statement is currently as follows: "All relevant data are within the manuscript and its Supporting Information files."

Please confirm at this time whether or not your submission contains all raw data required to replicate the results of your study. Authors must share the “minimal data set” for their submission. PLOS defines the minimal data set to consist of the data required to replicate all study findings reported in the article, as well as related metadata and methods (https://journals.plos.org/plosone/s/data-availability#loc-minimal-data-set-definition).

For example, authors should submit the following data:

- The values behind the means, standard deviations and other measures reported;

- The values used to build graphs;

- The points extracted from images for analysis.

Authors do not need to submit their entire data set if only a portion of the data was used in the reported study.

If your submission does not contain these data, please either upload them as Supporting Information files or deposit them to a stable, public repository and provide us with the relevant URLs, DOIs, or accession numbers. For a list of recommended repositories, please see https://journals.plos.org/plosone/s/recommended-repositories.

If there are ethical or legal restrictions on sharing a de-identified data set, please explain them in detail (e.g., data contain potentially sensitive information, data are owned by a third-party organization, etc.) and who has imposed them (e.g., an ethics committee). Please also provide contact information for a data access committee, ethics committee, or other institutional body to which data requests may be sent. If data are owned by a third party, please indicate how others may request data access.

6. If the reviewer comments include a recommendation to cite specific previously published works, please review and evaluate these publications to determine whether they are relevant and should be cited. There is no requirement to cite these works unless the editor has indicated otherwise.

[Note: HTML markup is below. Please do not edit.]

Reviewers' comments:

Reviewer's Responses to Questions

**Comments to the Author**

1. Is the manuscript technically sound, and do the data support the conclusions?

Reviewer #1: Yes

Reviewer #2: Yes

**********

2. Has the statistical analysis been performed appropriately and rigorously?

Reviewer #1: Yes

Reviewer #2: Yes

**********

3. Have the authors made all data underlying the findings in their manuscript fully available?

The PLOS Data policy

Reviewer #1: Yes

Reviewer #2: Yes

**********

4. Is the manuscript presented in an intelligible fashion and written in standard English?

Reviewer #1: Yes

Reviewer #2: Yes

**********

Reviewer #1: This is a clinically relevant study addressing the role of serum reverse triiodothyronine in dogs with hypothyroidism, nonthyroidal illness syndrome, and healthy controls. Overall, the study appears to be well designed and clearly presented; however, some points require clarification.

Line 16: Authors write that “tT4 is low in 30.2 – 52.5% of dogs with nonthyroidal illness syndrome (NTIS)”. This is not in agreement with the ALIVE criteria, “the syndrome is typically characterised by decreased circulating total T4 concentrations.”, therefore in all dogs and non only in some of them (Niessen et al. Agreeing Language in Veterinary Endocrinology (ALIVE): Hypothyroidism, Hyperthyroidism, (Euglycaemic) Diabetic Ketosis/Ketoacidosis, and Diabetic Remission-A Modified Delphi-Method- Based System to Create Consensus Definitions. Vet Sci. 2026 Jan 1;13(1):35.

Line 18: I suggest so replace “ can differentiate” with “can be helpful in differentiating”

Line 20: replace “an” with “a”

Line 22: “All dogs…was conducted”. This sentence is not necessary and can be removed

Lines 30-31”The ROC area under the curve for rT3 was 0.981 (95% CI = 0.951-1.010).”The sentence is incomplete; it should explain what the ROC curve was used for.

Line 36 and 37: “using HPLC-MS/MS”. This is not a conclusion, this sentence should be in the M&M

Line 43. I suggest to replace “diseases” with “conditions”

Lines 45-47 “Diagnosing hypothyroidism by tT4 analysis is challenging as tT4 is not only low in hypothyroidism, but also in 31% of dogs with nonthyroidal illness syndrome (NTIS).”See my comment above. Do not confuse non-thyroid disease with nonthyroidal illness syndrome. In the definition of nonthyroidal illness syndrome, the T4 is usually low

Line 123: “assessment of tT4” Indicate the analytical performance of the method for measuring tT4 and TSH, also indicating the limit of quantification of the method. Tables show tT4 values as low as 0.13 ng/dl. Is the method linear at those tT4 concentrations?

Lines 220-226: “Median rT3 concentration….in dogs with hypothyroidism and healthy controls” please report the data relating to fT4 in the same descriptive manner.

Discussion: The discussion is good. However, there are some studies in which rT3 has previously been evaluated in dogs. It would be appropriate to cite them. E.g. Wood et al. Influence of isoflurane general anesthesia or anesthesia and surgery on thyroid function tests in dogs.J Vet Intern Med. 2009 Jan-Feb;23(1):7-15. Giunti et al Retrospective evaluation of circulating thyroid hormones in critically ill dogs with systemic inflammatory response syndrome. J Vet Sci. 2017 Dec 31;18(4):471-477.

Reviewer #2: General comments

I would like to thank the authors for sharing these interesting and original data and compliment them on their work. The manuscript presents relevant data on the use of rT3, including validation of a new assay in dogs, definition of a reference interval, and a diagnostic accuracy study for hypothyroidism. However, in my opinion, many issues remain to be clarified, and the manuscript should be thoroughly revised.

Materials and Methods and the Results sections must be revised (see specific comments) to better adhere to the STARD guidelines for reporting diagnostic accuracy studies (https://www.equator-network.org/reporting-guidelines/stard/).

In the Abstract and Results sections, I strongly suggest adding effect estimates (e.g., median differences) with 95% confidence intervals for both statistically significant and non-significant results. This approach gives more precise information to the reader, allowing for a better interpretation of the results beyond the P value.

I suggest re-assessing the Discussion to better focus on the many results reported in the study, rather than reporting a general discussion of the pathophysiological mechanisms, which seems beyond the scope of the manuscript. Furthermore, some results require a more detailed discussion.

I also suggest reviewing the most recent literature, as many of the citations are outdated. Indeed, reported citations are appropriate, but integration with the most recent publications is desirable.

Regarding the literature search, I suggest defining how it was conducted. Rather than simply stating ‘there are no studies’ or ‘based on the author's knowledge’, it is preferable to add a line that defines how the search was performed (https://onlinelibrary.wiley.com/doi/10.1111/vru.12748).

For example, instead of lines 73-74 ‘Comparative studies on rT3 measurement in dogs are not currently available’, the authors can state ‘The following databases (XX, XX and XX) have been searched with the following keywords XX XX on [date, eg 01/01/2020], and no other reports of XX have been found’ or a similar formulation. This also allows the authors to modify the discussion. Whenever referencing in the Discussion (or any other section) to this, you can use statements like "Based on the literature search, this is the first time ..." or similar (see lines 254 and 273).

Specific comments

Abstract

Line 29: Median value is 2.5, while the lower limit of the range is <5. I am not sure if this is the correct way to report these results. If you define values <5 as equal to 2.5 for statistical reasons (line 162-163), you should probably report the lower limit of the range equal to 2.5. Please check and, if the same issue occurs, report it in the results section.

Line 32-33: Please add the confidence interval for the sensitivity and specificity.

Introduction

Line 46: When citing multiple studies, you should report the results of all these studies. There are different possible approaches. First, you can cite all the results (e.g., 31%, xx %, and xx %); second, you can just report the range (e.g., 31% to xx%, or xx% to 31%), as you did in line 65; finally, if you choose this percentage because it is the highest one reported, clearly state this (e.g., in up to 31%). Of note, you reported a range of prevalence in the abstract (line 16).

Line 47: I suggest revising this statement. Even though a clear definition of NTIS is difficult to derive solely from T4 measurements (which is the definition of NTIS?), it is generally accepted that low T4 is a criterion for defining the occurrence of NTIS in veterinary medicine, since we do not routinely measure T3. If not, all dogs with any disease should be considered as affected by NTIS. In this context, it is more appropriate to use the definition ‘non-thyroidal illness’ (without syndrome) or simply ‘concurrent diseases’, with the second one probably avoiding some confusion between ‘non-thyroidal illness’ and ‘non-thyroidal illness syndrome’.

Line 48: ‘Stimulation’ instead of ‘response’. Also, I guess the definition of "non-invasive" could be called into question. Indeed, thyroid scintigraphy is certainly demanding, but not necessarily invasive (see comments on line 53). I suggest using ‘gold standard among the blood tests’, or something similar.

Line 48-51: Please check the most recent literature: https://onlinelibrary.wiley.com/doi/10.1111/jsap.13290

Line 53: Is it always the case? It probably depends on the protocol used, but I guess that thyroid scintigraphy can also be performed under sedation.

Line 61: see comment on line 46 about the use of percentage extrapolated from the literature.

Line 58-63: Free T4-ED is not even mentioned among possible diagnostic tests. Since it is usually considered the most accurate test among the non-dynamic tests, I guess it is worth mentioning. It is even more important, given that the authors compared rT3 and fT4-ED in their study.

Line 66: Since rT3 is the core of the study, a brief description of rT3 is due before describing its use in humans and veterinary medicine. Furthermore, is it routinely used in humans?

I would consider scaling back the assumption about the usefulness of rT3 in humans: ‘has been suggested as a useful test to…’ instead of ‘is useful’?

Line 69-73: Please specify if total T3 (tT3) or free T3 (fT3).

Line 70: ‘Lower’ instead of ‘low’

Line 75: I suggest adding the validation of the assay as an aim of the study. It is an important result reported by the authors, but not adequately highlighted. Also, it seems that another aim of this study could be to compare rT3 and fT4-ED (see comment on lines 58-63).

Line 78: The study's aims should be followed by the author's hypothesis, at least for the comparative part of the study (e.g., We hypothesized that rT3 accurately…).

M&M:

The study design is not clearly stated. ‘Prospective’ is not a study design, simply describing how dogs were included time-wise. In my opinion, the study has two distinct parts that should be described appropriately without overlap; the authors did so in the results section but not in the MMs section. The first part involves assay validation and the definition of a reference interval; the second part of the study is a diagnostic accuracy study assessing the performance of rT3. I recommend consulting STARD guidelines for reporting diagnostic accuracy studies, https://www.equator-network.org/reporting-guidelines/stard/. The M&M section should be revised accordingly.

The main problem here is the definition of the reference standard: there is no specific test against which to compare rT3, but the proposed composite reference standard is somewhat acceptable from a clinical perspective. Still, this limitation should be clearly stated in the discussion, as you partially did in lines 336-340.

Line 86-90: The original population from which dogs were included should be better defined, as well as the process leading to the inclusion of dogs in the groups. For example, what were the criteria used to consider a dog a potentially eligible participant (e.g., all dogs presented in the selected period in which a thyroid panel was performed)? Have you decided a priori how many dogs to include in each group, or do you include all dogs that meet the appropriate criteria?

Related to this, it seems that healthy dogs were included differently from the dogs used in the diagnostic accuracy study.

Line 92: Pruritus is not a clinical sign of hypothyroidism. Indeed, it can be present if there is concurrent pyoderma, but it should not be included among clinical signs suggesting hypothyroidism.

Line 97-98: What does ‘moderate to severe’ underlying disease mean? Which criteria were used to define a disease as moderate to severe?

Furthermore, how was hypothyroidism ruled out in NTIS dogs showing low tT4 and high TSH?

Line 106: Whenever drug administration is used as an exclusion criterion, the time should be clearly stated. Was only ongoing treatment considered, or also previous treatment? In the second case, for how long had the drug to be discontinued before inclusion?

Line 108: Were TKIs included in the list of drugs leading to exclusion from the study? If not, can you please explain why?

Line 121: The commercial name of the profile is irrelevant; I would simply report the parameter measured in each dog (e.g, ‘complete blood count, serum chemistry profile including……, and total T4 and TSH were measured by Antech Lab’)

Line 130: Please specify the assays used to measure tT4 and TSH. I guess the lab used Siemens Immulite canine T4 and canine TSH, but it should be clearly stated. Also, specify if the reported intervals were calculated by the Lab.

Line 133-141: I would consider reporting this section as the results of the validation of the assay, rather than MM. These data are relevant, given that this assay was not previously validated in dogs (see line 75).

Line 143-151: It is not clear to me why all these parameters, except for FT-ED, were measured in both groups. Indeed, the results about tT3, T4AA, T3AA, and TgAA were not analyzed (not even descriptively) nor discussed. As it is, they seem quite out of context. Nonetheless, some of this data could probably be analyzed in an interesting way. For example, based on what is described in lines 66-69, has the author considered the possibility of analyzing the T3 to rT3 ratio in these dogs?

It clearly was not planned a priori, but the authors could conduct it as a post hoc analysis and appropriately report it in the Statistical Analysis section.

Line 154: Why were the data expressed as median and range? Was it due to non-normal distribution? How was the distribution of the data assessed? Furthermore, please be aware that range is not a measure of dispersion for non-normally distributed data; these should be described using other measures (e.g., the interquartile range). For this reason, IQR provides a better assessment of the variability of a dataset compared to range.

Line 156-157: I suggest to clearly state which method was used here, and not in the results (line 194-195). E.g., ‘…was calculated using a robust untransformed…based on the…’

Line 159: This sentence is not clear. The authors must describe here in the MM the analysis performed. E.g., ‘In healthy dogs, rT3 values were compared between young (healthy dogs <6 years) and old dogs (healthy dogs >/=7 years) using…’. Also, if the authors want to explore the correlation between age and rT3 values, it could be interesting to perform a correlation analysis (e.g. Spearman’s correlation).

Line 161: The ROC curve analysis was performed for both rT3 and FT4-ED, but the latter is not reported here. Please add it.

Line 164: See also the general comment of estimates of effects and confidence interval. Please add this part in the statistical section.

Results:

Line 167-168: I strongly suggest adding a flowchart at the beginning of this section, to highlight how the population in the diagnostic accuracy part of the study was created, as suggested by the STARD guidelines https://www.equator-network.org/wp-content/uploads/2015/03/STARD-2015-flow-diagram.pdf. Also, considering dogs were included from many centers, the number of dogs included in each center (Haar, University and lab-referred) should be clarified.

Line 176-181: Please avoid the use of ‘tends to’ or reporting population as younger or older if statistical significance is not achieved. To make these comparisons clearer, I suggest summarizing data about sex, age and body weight in a Table, describing only the comparisons with median difference and confidence intervals in the text. E.g., ‘No age difference was observed between hypo and healthy dogs (MD = xxx, 95% CI xx-xx, p= xxx) nor between NTIS and hypo dogs (MD = xxx, 95% CI xx-xx, p= xxx), while NTIS were older compared to healthy dogs (MD = xxx, 95% CI xx-xx, p= xxx),’. This way, the reader has all data to critically analyze the results reported, without the use of speculative terms such as ‘tends to’.

The same suggestions apply to all the Results section.

Figure 1: See previous comment. I suggest to graphically underline the significant differences between NTIS and healthy dogs.

Line 188: I would put the results of the validation here, along with the ones regarding the reference interval, in a dedicated paragraph. E.g., ‘Validation of rT3 assay and reference interval in healthy dogs.’

Table 1, 2, or 3 and lines 200-209: I suggest moving these three tables in the Supplementals. Even if the possibility to look at single dog is interesting, I do not believe it is really required in the main texts. Rather, I would summarize the descriptive analysis (median, IQR, range) regarding T4, TSH, rT3, and FT4 in the three groups in a single table, reporting the results of the comparisons in the text (see previous comments).

Furthermore, it could be interesting to know how many dogs (percentage) in hypo and NTIS group showed rT3 and fT4-ED values below (which means a positive test), within, or above (which means a negative test) the reference interval. In a diagnostic accuracy study, this is more valuable than a comparison between median values of healthy dogs and hypo/NTIS dogs: to extremize the concept, median values of dogs in hypo/NTIS groups could hypothetically be lower compared to healthy ones, but still, most of the single value of each dog could remain within the reference interval.

Figure 2: I suggest to graphically show the reference interval, to emphasize the distribution of the rT3 below, within or above the RI in the three groups.

Line 227: This sentence should be in the statistical analysis section.

Line 228-232 and 238-242: Please add the confidence interval for sensitivity and specificity values.

Figure 3 and 4: I would consider joining these two figures, to better emphasize the comparison between the ROC curves of the different tests.

Discussion:

Line 250-253: The incipit of the discussion usually summarizes the most relevant results of the study. In my opinion, I would add here the validation of the assay and the better performance of the rT3 compared with fT4-ED.

Line 254-255: The authors stated there are no comparative studies, but later they discuss the results of Corsini et al. 2025 (line 294-315). I agree with the authors that there are no studies published on peer-reviewed journals (provided their literature search confirm this assumption – see general comments), since Corsini et al. is a conference presentation and those results are unpublished to date. However, this discrepancy is not adequately discussed. I would probably consider at least underlining the nature of the Corsini at al. study in the manuscript.

Line 256-274: This entire paragraph is out of focus and should be removed from the discussion. This is merely a pathophysiological discussion about rT3 and NTIS, which is not related to the results reported by the authors. Indeed, the discussion must focus on the results presented in the study. This section could be moved, in a shorter and summarized version, in the Introduction to give the reader some information on what rT3 is and why it could be useful.

Line 276-277: see previous comments

Line 279-281: These are results, that should not be repeated in the discussion. Rather, discuss the implication of these results, if considered relevant.

Line 281-283: That’s true, but is this clinically relevant? We do not use any of this test to discriminate healthy and hypothyroid dogs, but rather to discriminate dogs with clinical signs (or laboratory abnormalities) due to hypothyroidism or due to NTIS.

Line 284-285: Please, go into details. For example, you should comment on the discrepancy in the percentage of dogs with NTIS with high rT3 (which must be added in the results, see previous comments) compared to humans. Which are the possible explanations? Maybe assessing tT3 and rT3 (see previous comments) can give you some idea.

Line 290-293: This seems to me the most interesting and clinically relevant result of the present study. I would highlight it and discuss it properly.

Line 317: ‘is’ rather than ‘could be explained’

Line 328-329: Please check the literature: https://academic.oup.com/jvim/article/38/3/1345/8456062

Line 332: It may be true, but it is not explained why the sample size limits the interpretation of the Results. For example, smaller samples lead to decreased statistical power; of note, this study has no power analysis, and this is a limitation worthy to remember. The (hypothetical) low statistical power increase the risk of type II error (failing to detect a difference that exist); however, does not seem the case considering the authors were able to demonstrate their hypothesis. Another possible limitation related to the low statistical power, is that the confidence intervals could be very wide; however, since now confidence intervals are not reported for most of the results, I am not able to comment on this (and so is the hypothetical reader).

Line 336-339: See previous comments, reported here - The main problem here is the definition of the reference standard: there is no specific test against which to compare rT3, but the proposed composite reference standard is somewhat acceptable from a clinical perspective.

Line 340-341: This is a result. The fact that rT3 was rechecked after treatment or after resolution of the concomitant diseases in a subset of dogs should be clearly described in the MM section/Study design, and these lines moved from the Discussion to the Results. Then, you can refer to the result to support you argumentation here.

Line 357-358: This is well discussed, but it could be even better to have a correlation analysis (see previous comments).

Line 361-364: Same comment as lines 250-253

Line 364-368: I would remove these lines. Generically stating the need of further studies is of no value. Rather, the authors could focus on the indication of performing more studies in dogs with different conditions or subclinical hypothyroidism, if they believe those are the settings that should be further investigated based on their results.

References

Line 472: This citation should be revised (https://doi.org/10.1093/jvimsj/aalaf018). Please check the guidelines for citing conference abstracts.

**********

what does this mean?). If published, this will include your full peer review and any attached files.

If you choose “no”, your identity will remain anonymous but your review may still be made public.

**Do you want your identity to be public for this peer review?** For information about this choice, including consent withdrawal, please see our Privacy Policy

Reviewer #1: **Yes:**  Federico Fracassi

Reviewer #2: **Yes:**  Andrea Corsini

**********

[NOTE: If reviewer comments were submitted as an attachment file, they will be attached to this email and accessible via the submission site. Please log into your account, locate the manuscript record, and check for the action link "View Attachments". If this link does not appear, there are no attachment files.]

To ensure your figures meet our technical requirements, please review our figure guidelines: https://journals.plos.org/plosone/s/figures

You may also use PLOS’s free figure tool, NAAS, to help you prepare publication quality figures: https://journals.plos.org/plosone/s/figures#loc-tools-for-figure-preparation.

NAAS will assess whether your figures meet our technical requirements by comparing each figure against our figure specifications.

Attachment Submitted filename: Revisione_repeated thyroid function.docx

# Author comment: Author response to Decision Letter 1

**DOI:** 10.1371/journal.pone.0357106.r002

27 May 2026

Dear Editor and Reviewers,

We would like to thank you for the constructive feedback on our manuscript. We have carefully addressed all comments and questions raised by the editor and reviewers. A detailed point-by-point response to all comments has been provided in our attached 'Response to Reviewers' document, and the manuscript has been revised accordingly.

Sincerely,

Julia Ruß

Attachment Submitted filename: Response to Reviewers.docx

# Peer review: Decision Letter 1

**DOI:** 10.1371/journal.pone.0357106.r003

**Authors:** Benito Soto-Blanco

30 Jun 2026

Dear Dr. Ruß,

Thank you for submitting your manuscript to PLOS ONE. After careful consideration, we feel that it has merit but does not fully meet PLOS ONE’s publication criteria as it currently stands. Therefore, we invite you to submit a revised version of the manuscript that addresses the points raised during the review process.

Please submit your revised manuscript by Aug 14 2026 11:59PM. If you will need more time than this to complete your revisions, please reply to this message or contact the journal office at plosone@plos.org. When you're ready to submit your revision, log on to https://www.editorialmanager.com/pone/ and select the 'Submissions Needing Revision' folder to locate your manuscript file.

- A letter that responds to each point raised by the academic editor and reviewer(s). You should upload this letter as a separate file labeled 'Response to Reviewers'.
- A marked-up copy of your manuscript that highlights changes made to the original version. You should upload this as a separate file labeled 'Revised Manuscript with Track Changes'.
- An unmarked version of your revised paper without tracked changes. You should upload this as a separate file labeled 'Manuscript'.

If you would like to make changes to your financial disclosure, please include your updated statement in your cover letter. Guidelines for resubmitting your figure files are available below the reviewer comments at the end of this letter.

If applicable, we recommend that you deposit your laboratory protocols in protocols.io to enhance the reproducibility of your results. Protocols.io assigns your protocol its own identifier (DOI) so that it can be cited independently in the future. For instructions see: https://journals.plos.org/plosone/s/submission-guidelines#loc-laboratory-protocols. Additionally, PLOS ONE offers an option for publishing peer-reviewed Lab Protocol articles, which describe protocols hosted on protocols.io. Read more information on sharing protocols at https://plos.org/protocols?utm_medium=editorial-email&utm_source=authorletters&utm_campaign=protocols.

As the corresponding author, your ORCID iD is verified in the submission system and will appear in the published article. PLOS supports the use of ORCID, and we encourage all coauthors to register for an ORCID iD and use it as well. Please encourage your coauthors to verify their ORCID iD within the submission system before final acceptance, as unverified ORCID iDs will not appear in the published article. *Only* the individual author can complete the verification step; PLOS staff *cannot* verify ORCID iDs on behalf of authors.

We look forward to receiving your revised manuscript.

Kind regards,

Benito Soto-Blanco, DVM, MSc, PhD

Academic Editor

PLOS One

Journal Requirements:

If the reviewer comments include a recommendation to cite specific previously published works, please review and evaluate these publications to determine whether they are relevant and should be cited. There is no requirement to cite these works unless the editor has indicated otherwise.

Please review your reference list to ensure that it is complete and correct. If you have cited papers that have been retracted, please include the rationale for doing so in the manuscript text, or remove these references and replace them with relevant current references. Any changes to the reference list should be mentioned in the rebuttal letter that accompanies your revised manuscript. If you need to cite a retracted article, indicate the article’s retracted status in the References list and also include a citation and full reference for the retraction notice.

[Note: HTML markup is below. Please do not edit.]

Reviewers' comments:

Reviewer's Responses to Questions

**Comments to the Author**

Reviewer #2: (No Response)

**********

2. Is the manuscript technically sound, and do the data support the conclusions?

Reviewer #2: Yes

**********

3. Has the statistical analysis been performed appropriately and rigorously?

Reviewer #2: Yes

**********

4. Have the authors made all data underlying the findings in their manuscript fully available?

The PLOS Data policy

Reviewer #2: Yes

**********

5. Is the manuscript presented in an intelligible fashion and written in standard English?

Reviewer #2: Yes

**********

Reviewer #2: The authors adequately responded to many of my concerns. However, I have some further comments that I would like to see addressed before publication.

I previously commented on the use of point estimates of effect (mean or median difference) with 95% CI as a tool to better describe their results, providing more information to the reader. However, I think the authors may have misunderstood my comment. Indeed, estimates of effect were not included (https://www.acpjournals.org/doi/10.7326/0003-4819-114-6-515 or https://www.bmj.com/content/292/6522/746), although the authors stated that they had done so for “age”. Instead, the authors reported 95% CI together with the mean. The use of mean +/- 95% CI is possible, but not for describing the variability in the population from which the sample was drawn (see: https://doi.org/10.1136/bmj.331.7521.903), which is the usual purpose of descriptive statistics. This is also the rationale for using IQR when reporting medians. I suggest describing “weight” using mean ± standard deviation, which seems more useful than the standard error and derived variables, such as confidence intervals, in this specific setting.

Line 52: The AUC for fT4d is already reported in line 49. Please remove one of the two istances. I would report the exact value corresponding to the lower limit of the RI for fT4d.

Line 70: The term NTIS is used for the first time here, but it is not spelled out. This also occurs several times before line 105, in which the author spelled out NTIS. Also, NTI is still used to denote diseases not affecting the thyroid that could influence thyroid function; even if the distinction from NTIS is clearer and the use of NTI is appropriate, I believe that using a different term would help avoid any possible confusion between the syndrome leading to low T4 (NTIS) and concurrent non-thyroidal diseases (NTI).

Line 105: I suggest using ‘humans’ instead of ‘men’, as the latter could be misleading and interpreted as referring specifically to the male sex.

Line 110: A paper validating rT3 measured with LC-MS/MS assay in healthy dogs has recently been published (https://doi.org/10.1016/j.domaniend.2026.107024). I believe it would be worth citing and discussing this study. For example, the reported reference interval (107–542 pg/mL) appears very similar to that described in the present study, despite LC-MS/MS being performed in two different laboratories. This seems promising, as it may suggest low inter-assay variability, particularly at the lower cut-offs.

Line 197-198: It is commendable that the authors performed an a priori statistical power analysis to justify the target sample size. However, the power analysis itself should be described in sufficient detail in the statistical section. Specifically, the authors should report the assumed effect size, significance level (α), desired power (1 − β), statistical test used, and the software employed to perform the calculation.

Lines 306-308: Please clarify the rationale for excluding the three outliers. Were they identified using a predefined statistical outlier detection method or excluded for analytical/clinical reasons? Furthermore, the statement that the selected reference interval "provided the most plausible results" is subjective and should be replaced with an objective description of the statistical method used to derive the final reference interval.

Lines 321–345: I would consider reporting the sensitivity and specificity results for different cut-offs in a table. This would improve the interpretation and comparison of the different cut-offs.

Table 4: I think this table would be more informative and useful if the pairwise comparisons were reported as median differences with 95% confidence intervals instead of p-values.

Table 5: Are the authors sure that the term “negative test” is appropriate for results above the reference interval? Indeed, in clinical practice, a normal fT4ed or rT3 result is usually considered “negative”.

**********

what does this mean?). If published, this will include your full peer review and any attached files.

If you choose “no”, your identity will remain anonymous but your review may still be made public.

**Do you want your identity to be public for this peer review?** For information about this choice, including consent withdrawal, please see our Privacy Policy

Reviewer #2: **Yes:**  Andrea Corsini

**********

[NOTE: If reviewer comments were submitted as an attachment file, they will be attached to this email and accessible via the submission site. Please log into your account, locate the manuscript record, and check for the action link "View Attachments". If this link does not appear, there are no attachment files.]

To ensure your figures meet our technical requirements, please review our figure guidelines: https://journals.plos.org/plosone/s/figures

You may also use PLOS’s free figure tool, NAAS, to help you prepare publication quality figures: https://journals.plos.org/plosone/s/figures#loc-tools-for-figure-preparation.

NAAS will assess whether your figures meet our technical requirements by comparing each figure against our figure specifications.

# Author comment: Author response to Decision Letter 2

**DOI:** 10.1371/journal.pone.0357106.r004

10 Aug 2026

We thank the Academic Editor and Reviewer #2 for their careful and constructive evaluation of our manuscript.

We have addressed all comments and suggestions raised during the review process. A detailed point-by-point response to each comment is provided in the separately uploaded document entitled “Response to Reviewers,” where all corresponding changes in the revised manuscript are described and, where applicable, indicated by line numbers.

We have also carefully revised the manuscript for clarity, consistency, statistical reporting, and language, and have updated the relevant references as appropriate.

Regarding ORCID, we have asked all co-authors to register and/or verify their ORCID iDs in the PLOS ONE submission system. ORCID registrations are already available for Dr. Christina Schwensn, Prof. Ralf S. Mueller, and Stefanie Klenner-Gastreich in the PLOS ONE system. We have encouraged the remaining co-authors to complete their ORCID registration and verification prior to final acceptance.

We have uploaded the revised manuscript, the manuscript with tracked changes, and the detailed response to the reviewers as separate files, as requested.

Attachment Submitted filename: Response to Reviewers Part 2.docx

# Editor report: Decision Letter 2

**DOI:** 10.1371/journal.pone.0357106.r005

**Authors:** Benito Soto-Blanco

13 Aug 2026

Serum reverse triiodothyronine in dogs with hypothyroidism, nonthyroidal illness syndrome and healthy dogs

PONE-D-26-03433R2

Dear Dr. Ruß,

We’re pleased to inform you that your manuscript has been judged scientifically suitable for publication and will be formally accepted for publication once it meets all outstanding technical requirements.

Within one week, you’ll receive an e-mail detailing the required amendments. When these have been addressed, you’ll receive a formal acceptance letter and your manuscript will be scheduled for publication.

An invoice will be generated when your article is formally accepted. Please note, if your institution has a publishing partnership with PLOS and your article meets the relevant criteria, all or part of your publication costs will be covered. Please make sure your user information is up-to-date by logging into Editorial Manager at Editorial Manager® and clicking the ‘Update My Information' link at the top of the page. For questions related to billing, please contact billing support.

If your institution or institutions have a press office, please notify them about your upcoming paper to help maximize its impact. If they’ll be preparing press materials, please inform our press team as soon as possible -- no later than 48 hours after receiving the formal acceptance. Your manuscript will remain under strict press embargo until 2 pm Eastern Time on the date of publication. For more information, please contact onepress@plos.org.

Kind regards,

Benito Soto-Blanco, DVM, MSc, PhD

Academic Editor

PLOS One

# Editor report: Acceptance letter

**DOI:** 10.1371/journal.pone.0357106.r006

**Authors:** Benito Soto-Blanco

PONE-D-26-03433R2

PLOS One

Dear Dr. Ruß,

I'm pleased to inform you that your manuscript has been deemed suitable for publication in PLOS One. Congratulations! Your manuscript is now being handed over to our production team.

At this stage, our production department will prepare your paper for publication. This includes ensuring the following:

* All references, tables, and figures are properly cited

* All relevant supporting information is included in the manuscript submission,

* There are no issues that prevent the paper from being properly typeset

You will receive further instructions from the production team, including instructions on how to review your proof when it is ready. Please keep in mind that we are working through a large volume of accepted articles, so please give us a few days to review your paper and let you know the next and final steps.

Lastly, if your institution or institutions have a press office, please let them know about your upcoming paper now to help maximize its impact. If they'll be preparing press materials, please inform our press team within the next 48 hours. Your manuscript will remain under strict press embargo until 2 pm Eastern Time on the date of publication. For more information, please contact onepress@plos.org.

You will receive an invoice from PLOS for your publication fee after your manuscript has reached the completed accept phase. If you receive an email requesting payment before acceptance or for any other service, this may be a phishing scheme. Learn how to identify phishing emails and protect your accounts at https://explore.plos.org/phishing.

If we can help with anything else, please email us at customercare@plos.org.

Thank you for submitting your work to PLOS One and supporting open access.

Kind regards,

PLOS One Editorial Office Staff

on behalf of

Dr. Benito Soto-Blanco

Academic Editor

PLOS One
